# Characterization of the cholesterol biosynthetic pathway in *Dioscorea transversa*

**DOI:** 10.1016/j.jbc.2023.104768

**Published:** 2023-05-02

**Authors:** Lauren J. Salisbury, Stephen J. Fletcher, Jeanette E. Stok, Luke R. Churchman, Joanne T. Blanchfield, James J. De Voss

**Affiliations:** School of Chemistry and Molecular Biosciences, University of Queensland, Brisbane, Queensland, Australia

**Keywords:** cholesterol biosynthesis, CYP51, phytosterol biosynthesis, saponin, transcriptome

## Abstract

Cholesterol is the precursor of bioactive plant metabolites such as steroidal saponins. An Australian plant, *Dioscorea transversa*, produces only two steroidal saponins: 1β-hydroxyprotoneogracillin and protoneogracillin. Here, we used *D. transversa* as a model in which to elucidate the biosynthetic pathway to cholesterol, a precursor to these compounds. Preliminary transcriptomes of *D. transversa* rhizome and leaves were constructed, annotated, and analyzed. We identified a novel sterol side-chain reductase as a key initiator of cholesterol biosynthesis in this plant. By complementation in yeast, we determine that this sterol side-chain reductase reduces Δ^24,28^ double bonds required for phytosterol biogenesis as well as Δ^24,25^ double bonds. The latter function is believed to initiate cholesterogenesis by reducing cycloartenol to cycloartanol. Through heterologous expression, purification, and enzymatic reconstitution, we also demonstrate that the *D. transversa* sterol demethylase (CYP51) effectively demethylates obtusifoliol, an intermediate of phytosterol biosynthesis and 4-desmethyl-24,25-dihydrolanosterol, a postulated downstream intermediate of cholesterol biosynthesis. In summary, we investigated specific steps of the cholesterol biosynthetic pathway, providing further insight into the downstream production of bioactive steroidal saponin metabolites.

The most abundant sterols in plants are the C24-alkyl phytosterol group such as campesterol (**1**) and β-sitosterol (**2**, Fig. 1). However, a significant number of important secondary metabolites such as steroidal saponins (1) and glycoalkaloids (2) lack this C24-alkyl moiety, and this strongly implicates cholesterol (**3**) as their likely precursor. Biosynthesis of the C24-alkyl phytosterols begins with the 30-carbon precursor 2,3-oxidosqualene, which is cyclized by cycloartenol synthase (CAS) to yield cycloartenol (**4**, Fig. 1). Immediately following this, a C24-alkyl group is installed by a sterol methyl transferase (SMT) to generate **5**. This means that cholesterol (**3**) biosynthesis must diverge before this occurs. Cholesterol (**3**) is usually present only in low amounts in most plants. However, some *Dioscorea* are known to contain an abundance of bioactive steroidal saponins, which comprise up to 2% of the plant’s dry weight in some species (1).

**Figure 1:**
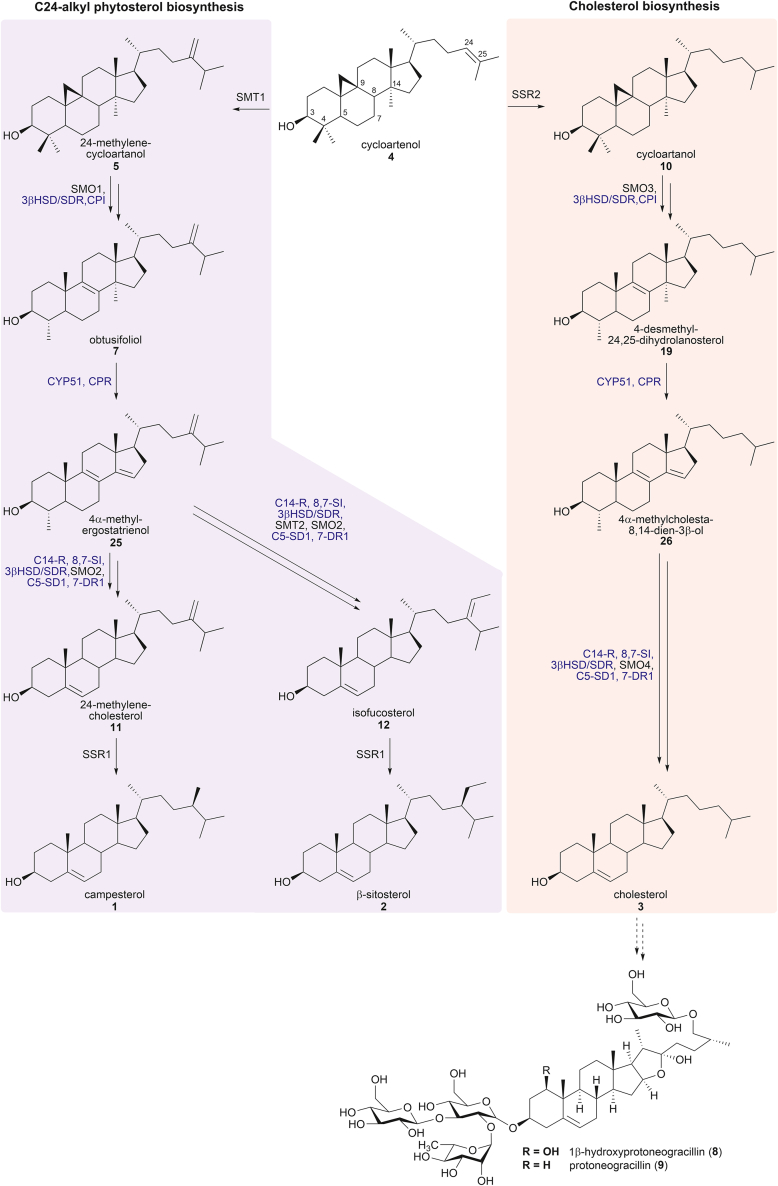
**The C24-alkyl phytosterols such as campesterol (1) and β-sitosterol (2) are the predominate sterols in plants.** However, a significant number of secondary metabolites are derived from cholesterol (**3**) such as 1β-hydroxyprotoneogracillin (**8**) and protoneogracillin (**9**) isolated from *Dioscorea transversa* (1). In *pink* is the putative cholesterol (**3**) biosynthetic pathway of the *Solanaceae* family. Evidence suggests that some plants generate cholesterol (**3**) from cycloartenol (**4**). In *Solanaceae*, it is believed that an SSR (SSR2) initiates the bifurcation of the phytosterol and cholesterol biosynthetic pathways by reducing cycloartenol (**4**) to cycloartanol (**10**), thus preventing methylation by SMT1 (6). Some of the enzymes (*black text*) involved appear to be specific to the phytosterol or to the novel cholesterol pathway, whereas others (*blue text*) are shared between both. 8,7-SI, sterol 8,7 isomerase; 3βHSD, 3β-hydroxysteroid dehydrogenase; C5-SD, sterol C-5(6)-desaturase 2; CPI, cyclopropylsterol isomerase; C14-R, sterol C-14 reductase; CYP51, sterol 14α-demethylase; 7-DR, 7-dehydrocholesterol reductase; SMO, sterol methyl oxidase; SMT, sterol methyl transferase; SSR, sterol side-chain reductase.

Whilst the cholesterol (**3**) biosynthetic pathway in animals has been defined for decades (3), plant cholesterogenesis is still somewhat enigmatic. In animals, cholesterol (**3**) biosynthesis is initiated by the lanosterol synthase (LSS) catalyzed cyclization of 2,3-oxidosqualene to generate lanosterol (**6**) (4). Some plants, such as *Arabidopsis thaliana* and members of the *Solanum* genus, express LSS alongside CAS, which could permit cholesterogenesis *via* lanosterol (**6**) (2). However, gene-knockout studies in these plants have revealed that LSS does not play a significant role in cholesterogenesis, and instead, an alternate pathway must yield cholesterol (**3**), presumably from cycloartenol (**4**) (5).

One such pathway has been tentatively defined in *Solanum lycopersicum*. Members of the *Solanum* genus such as *S*. *tuberosum* (potato) and *S*. *lycopersicum* (tomato) generate steroidal glycoalkaloids, which, like steroidal saponins, are derived from cholesterol (**3**) (2). In a gene-knockout study of *S. tuberosum*, it was found that this species may bifurcate its phytosterol metabolic pathway at cycloartenol (**4**), where the C24 of this precursor is either methylenated by SMT1 to initiate phytosterol biosynthesis or reduced by a unique and regioselective sterol side-chain reductase 2 (SSR2) to permit cholesterogenesis (Fig. 1) (2). A later study putatively defined eight additional transformations that follow reduction by SSR2 to yield cholesterol (**3**) (6). From a combination of gene-knockout and biochemical studies, four of these subsequent reactions are reportedly performed by enzymes, which also participate in phytosterol metabolism concurrently, postulating that the enzymes exhibit substrate promiscuity for intermediates in both pathways (6). These enzymes are present as a single copy in the genome of *S. lycopersicum*, and silencing them decreases the level of all sterols in the plant. For example, a single CYP51 enzyme within this plant appears to be responsible for the key demethylation of obtusifoliol (**7**) in phytosterol biosynthesis as well a potential intermediate in cholesterol biosynthesis. In contrast, the remaining four transformations are believed to be performed by unique enzymes that have presumably evolved from phytosterol biosynthetic enzymes to be specific for cholesterogenesis. Silencing these genes prevented cholesterol (**3**) formation, but phytosterols were still generated in similar quantities (6). As the phytosterol and cholesterol (**3**) pathways share a common precursor in *Solanum*, some mechanism must exist to control the metabolic flux through the cholesterol (**3**) biosynthetic pathway relative to phytosterol biosynthesis. One control mechanism previously postulated is through modulation of SMT1 expression levels, where a decrease in SMT1 expression favors cholesterogenesis (6). However, in *S. tuberosum*, SMT1 mRNA expression levels did not change appreciably; and instead increasing SSR2 expression favored cholesterogenesis by regioselective reduction of the Δ^24,^^25^ double bond of cycloartenol (**4**) (2).

The Australian native yam *D*. *transversa* (“Long yam” or “Kowar”), traditionally used mainly as a foodstuff (7), provides an ideal system in which to study plant-derived cholesterol (**3**) as it possesses an unusually simple steroidal saponin profile. The tubers contain only two saponins, the furostanols 1β-hydroxyprotoneogracillin (**8**) and protoneogracillin (**9**), at high concentration (Fig. 1) (1). As the first step in exploring steroidal saponin biosynthesis, we set out in this work to define the cholesterogenesis pathway in *D. transversa via* a combination of transcriptomics alongside *in vitro* and *in vivo* biochemical characterization of two key enzymes in the pathway, a CYP51 and an SSR.

## Results

### Transcriptome assembly and functional annotation

A transcriptome of *D. transversa* was constructed to identify potential enzymes in the cholesterol (**3**) biosynthetic pathway. Many, but not all, secondary metabolites are stored in the same location in which they are originally synthesized (8). Previous work with steroidal saponins from *D. zingiberensis* suggested that these secondary metabolites are both synthesized and stored in the tuber/rhizome of this plant rather than in the leaves (9). Hence, it was anticipated that *D. transversa* would behave in a similar fashion. As the steroidal saponins 1β-hydroxyprotoneogracillin (**8**) and protoneogracillin (**9**) were isolated from the rhizome of the plant (1), total RNA was extracted from the rhizome for the construction of the transcriptome and from the leaves for use as a transcriptome control. In addition, extraction of the steroidal saponins from the rhizome was performed to ensure that the tissue used to provide the total RNA contained steroidal saponins at the time of extraction. A portion of the same sample used to obtain the total RNA was extracted with 80% aqueous methanol and analyzed by HPLC and mass spectrometry (MS) confirming that this tissue was indeed producing both saponins (1). The total RNA from both the rhizome and the leaves was then used to generate a complementary DNA (cDNA) library that was sequenced on an Illumina HighSeq platform (Australian Genome Research Facility [AGRF]). The two libraries (rhizome and leaves) were sequenced on a single lane to produce 113,390,389 paired-end reads for the leaf sample and 116,072,936 paired-end reads for the rhizome sample. These sequenced libraries were used to construct a *de novo* assembly of the transcriptome using Trinity (https://github.com/trinityrnaseq/trinityrnaseq) (10). A total of 108,310 transcript isoforms (66,562 unigenes) were assembled, with 48,886 of these transcripts having ORFs. The assembled transcriptome had an N50 of 1915 bp and an average transcript length of 1147 bp, indicating many transcripts were nonfragmented and likely to be of full length. Other *Dioscorea* transcriptomes have reported similar transcript statistics: *D. zingiberensis*, 56,993 unigenes with an average length of 1142 bp (9); *D. alata*, 60,020 unigenes with an average length of 592 bp (11); and *D. composita*, 62,341 unigenes with an average length of 1368 bp (12).

Upon analysis of the transcriptome, no transcript corresponding to an LSS was observed indicating that *D. transversa* must not generate cholesterol (**3**) from its usual precursor lanosterol (**6**). In addition, a single CAS (TR2_c1_g1) was present and expressed in both the leaf (L) and rhizome (R) tissue (38:104 L:R transcripts per million [TPMs], Table S1). This suggested that cycloartenol (**4**) was the initial precursor of both the phytosterol and cholesterol (**3**) biosynthetic pathway(s) (Fig. 1). Overlap between these biosynthetic pathways has previously been proposed following transcriptome analysis of *D. zingiberensis* (9) and experimentally observed in *Solanaceae* (6). Thus, candidates for the enzymes involved in the biosynthesis of phytosterols and cholesterol (**3**) from cycloartenol (**4**) were sought. Homologs with high identity (ID) and similarity (SIM) to 11 of the 12 enzymes proposed for the transformation of cycloartenol (**4**) into cholesterol (**3**) (10 steps, Fig. 1) were identified (6), with the single missing homolog being a second SSR. The additional SMTs required for the generation of the C24-alkyl sterols *via* **5** were also observed in the transcriptome (Table S1). In some cases (SSR, 3βHSD, CPI, CYP51, cytochrome P450 reductase [CPR], and 7-DR), only a single transcript was found for the steps between cycloartenol (**4**) and the later steroids (**1–3**, Fig. 1), implying enzyme functionality in both the cholesterol (**3**) and phytosterol biosynthetic pathways. In other cases (C14-R, 8,7-SI, and C5SD1), duplicates were detected, and this suggests that these transcripts encode enzymes that may be specific for either the biosynthesis of cholesterol (**3**) or the C24-alkyl phytosterols. This type of gene duplication/apparent substrate promiscuity has previously been observed in the tomato *S. lycopersicum* (6).

### Identification of candidate SSR gene

**Figure 2:**
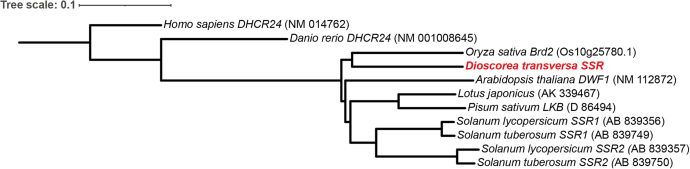
**Phylogenetic tree of homologous genes to *D***. ***transversa* sterol side-chain reductase (SSR*Dt*).** Sequence alignments of homologous transcripts were carried out using Clustal Omega and phylogenetic tree generation using Simple Phylogeny, with the clustering method set to “neighbor joining” and “distance correction” selected. The phylogenetic tree plot was generated by iTOL with the *Homo sapiens DHCR24* as the root.

One significant difference between the transcriptomes reported for members of the *Solanum* genus and that of *D. transversa* is that only a single SSR homolog (SSR*Dt*) is expressed in the latter (2, 6). In *S. lycopersicum* and *S. tuberosum*, cholesterogenesis is thought to be initiated by the chemospecific reduction of cycloartenol (**4**) to cycloartanol (**10**) by a unique SSR (SSR2) that is specific to the cholesterol (**3**) pathway (2, 6). However, only one complete SSR homolog was expressed in *D. transversa* that shared significant homology to SSR1 from *A. thaliana* (80% amino acid ID and 090% SIM). A second transcript also shared homology with SSR1 of *A. thaliana* (74% ID and 87% SIM), but the complete gene was not obtained (326 residues out of 560), and the transcript has low expression levels, especially in the rhizome where we postulated cholesterogenesis occurs (0.5:0 L:R TPM). Little functional information could be obtained from a phylogenetic analysis of SSR*Dt;* a phylogram showing the gene is simply clustered with the related monocotyledonous *Oryza sativa* (Fig. 2). This is distant from both the aforementioned Δ^24,^^25^ reductases (*S. lycopersicum SSR2* and *S. tuberosum SSR2*) and Δ^24,28^ reductases (*S. lycopersicum SSR1*, *S. tuberosum SSR1*, and *A. thaliana DWF1*).

If *D. transversa* generates **3** in a similar manner to that which is postulated for *S. lycopersicum*, it would need to be initiated by a multifunctional SSR, which can catalyze the reduction of the Δ^24,^^25^ double bond of cycloartenol (**4**) as well as reduction of the Δ^24,^^28^ double bond of 24-methylenecholesterol (**11**) and isofucosterol (**12**) in phytosterol formation. This dual function implies the need for a mechanism to control the metabolic flux between these pathways distinct from the regiospecific SSR2 proposed for *Solanum* spp. Our SSR*Dt* transcript was preferentially expressed in the rhizome (39:359 L:R TPM), and it is possible that the significant increase in relative mRNA expression level permits rapid reduction of the Δ^24,^^25^ olefin of cycloartenol (**4**). Lower-level expression in the leaves would allow methylation by SMT1 to be competitive with SSR reduction and allow phytosterol production to predominate. We observed that the SMT1 candidate (TR37901_c0_g1) exhibited similar expression levels in both tissues, suggesting that the metabolic flux may be controlled by differential expression of SSR*Dt*. If so, SSR*Dt* must be a promiscuous and multifunctional catalyst capable of reducing both cycloartenol (**4**) and advanced precursors of the phytosterols **1** and **2**, such as **11** and **12**.

### *In vivo* enzymatic activity assays of SSR

To investigate the reduction activities of SSR*Dt*, the confirmed genetic sequence was codon optimized and overexpressed in several strains of *Saccharomyces cerevisiae* (yeast), which featured mutations in the well-characterized biosynthetic pathway leading to ergosterol (**13**), the major sterol in this organism. Within this pathway, *erg4* encodes a Δ^24, 28^ SSR, which generates ergosterol (**13**) from Δ^24,^^28^ substrate ergosta-5,7,22,24(28)-tetraenol (**14**) and accordingly *erg4*Δ yeast accumulates **14** (13). The activity of ERG4 is therefore analogous to one of the predicted functions of SSR*Dt*: reduction of Δ^24,^^28^ in 24-methylenecholesterol (**11**) and isofucosterol (**12**) in *D. transversa*. Thus, observing complementation of ERG4 by SSR*Dt* by analyzing the sterol profile of a complemented ERG4 mutant would allow us to confirm the role of this enzyme in phytosterol metabolism.

ERG6 in yeast is an SMT, which methylates C24 of zymosterol. Yeasts that are deficient in this gene accumulate variants of the ergosterol (**13**) pathway intermediates, which retain the Δ^24,^^25^ double bond, such as zymosterol (13). Consequently, it was envisaged that overexpressing SSR*Dt* in *erg6*Δ yeast would reveal whether this enzyme is multifunctional as predicted and able to reduce sterol Δ^24,^^25^ double bonds, such as that found in cycloartenol (**4**) in the predicted cholesterol (**3**) biogenesis (Fig. 1).

**Figure 3:**
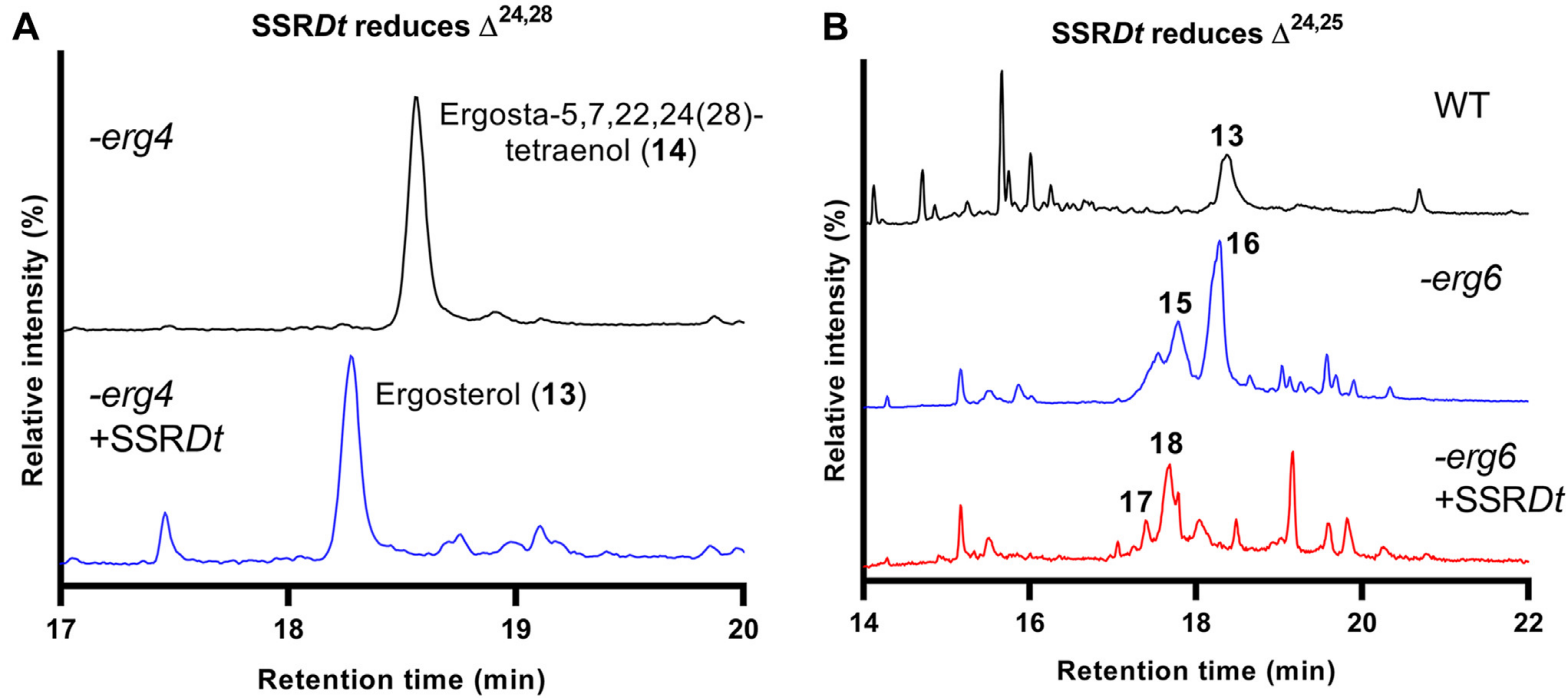
**GCMS analysis of extracted sterols from yeast***.A*, in *erg4*Δ yeast (*top trace*), the sterol (**14**) possessing a Δ^24,^^28^ double bond accumulated. When SSR*Dt* was expressed in *erg4*Δ yeast (*bottom*), the organism converted this metabolite (**14**) into ergosterol (**13**), indicating that SSR*Dt* was reducing the Δ^24,^^28^ double bond of (**14**). *B*, the *erg6*Δ yeast (*middle trace*) generated sterols, which featured a Δ^24,^^25^ unsaturation in the steroid side chain (**15, 16**) not previously observed in WT (*top*), which produced mainly ergosterol (**13**). When SSR*Dt* was expressed in this yeast (*bottom*), the metabolites **17** and **18** lacked this unsaturation indicating that SSR*Dt* was reducing the Δ^24,^^25^ double bonds. The fragmentation patterns of **13** to **18** can be found in Figs. S1–S4. SSR*Dt*, *D*. *transversa* sterol side-chain reductase.

Both the *erg4*Δ and *erg6*Δ yeast mutants were separately generated by subcloning a HISMX gene fragment to disrupt each gene. Following this, a pRS426GPD plasmid was manipulated to carry SSR*Dt*, and the construct was subsequently cloned into each yeast mutant. The cells were lysed, and after organic extraction, metabolites were analyzed by GC–MS. The major sterols (Fig. 3) in each case were identified by comparison of the mass spectra with known metabolites of the WT, *erg4*Δ, and *erg6*Δ yeast or to authentic standards where possible. As expected, the *erg4*Δ yeast did not generate any ergosterol (**13**), and instead, the major sterol accumulated by the yeast was identified by GC–MS analysis as **14** (Fig. 3*A*). When SSR*Dt* was expressed in this mutant, ergosterol (**13**) was present as the major sterol in the extract, indicating that the SSR complemented the missing ERG4 activity. From this, it is clear that SSR*Dt* is capable of reducing the Δ^24,^^28^ alkene required to generate campesterol (**1**) and β-sitosterol (**2**) in phytosterol biosynthesis (Fig. 1).

**Figure 4:**
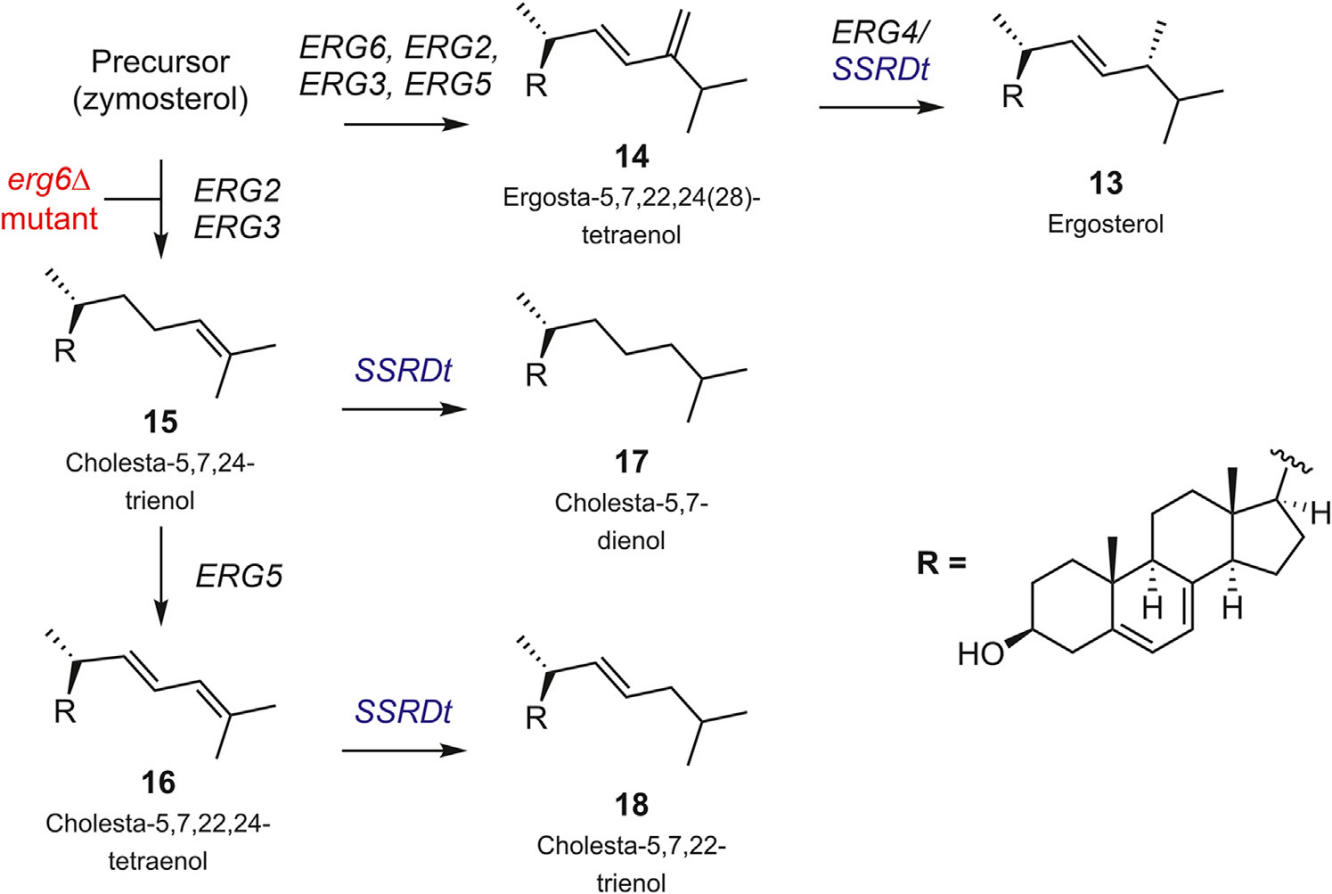
**Schematic of sterol side-chain formation in *Saccharomyces cerevisiae* including the reactions catalyzed by SSR*Dt* (*blue*) when this enzyme is heterologously expressed in the organism.** In *erg4*Δ yeast, SSR*Dt* fulfils the same role as ERG4 to reduce the 24(28) double bond of **14**, the major sterol seen in this mutant. In *erg6*Δ yeast (*red*), SSR*Dt* is capable of reducing the 24(25) double bond of **15** and **16**, the major sterols of this mutant. SSR*Dt*, *Dioscorea transversa* sterol side-chain reductase.

Disruption of *erg6* resulted in the generation of a complex mix of sterols (Fig. 3*B*). Through careful analysis of the fragmentation pattern and comparison with known metabolites of *erg6*Δ yeast (13, 14), we were able to identify two major components of the profile (**15**, **16**), which possessed a Δ^24,^^25^ unsaturation. Upon expression of SSR*Dt* in this mutant, a complete change in the steroid profile occurred (Fig. 3*B*). None of the steroids previously identified in the *erg6*Δ yeast were detected. Analysis of the fragmentation pattern of two peaks led to the tentative identification of two new metabolites (**17, 18**). The fragmentation patterns of these compounds correlate with loss of the Δ^24,^^25^ unsaturation observed for the *erg6*Δ yeast metabolites earlier, indicating that SSR*Dt* is reducing the Δ^24,^^25^ double bond on the sterol side chain. From this, it can be concluded that SSR*Dt* is a promiscuous and multifunctional enzyme capable of reducing both sterol Δ^24,^^28^ and Δ^24,^^25^ double bonds (Fig. 4).

### Identification of candidate genes: CYP51*Dt* and CPR*Dt*

Having shown that variation in SSR level of expression was a potential mechanism by which *D. transversa* controlled cholesterol (**3**) biosynthesis relative to that of C24-alkyl phytosterols, overlap between these two pathways was investigated. One of the key steps in the biosynthesis of all steroids is the 14α-demethylation reaction catalyzed by a cytochrome P450 sterol, 14α-demethylase (CYP51). This ancient enzyme family is found in all kingdoms of life, and its function is almost exclusively this key demethylation step in sterol biosynthesis with only a single known exception (15). Usually, this activity is limited to lanosterol (**6**) or its analogs as precursors in cholesterol (**3**) biosynthesis and obtusifoliol (**7**) and its analogs as precursors for phytosterol production.

However, in gene-knockout studies, Δ*cyp51* mutants of *S. lycopersicum* (6) accumulated obtusifoliol (**7**), the classical plant CYP51 substrate, as well as 4-desmethyl-24,25-dihydrolanosterol (**19**), a possible intermediate for cholesterol (**3**) production (Fig. 1). As such, it is believed that the CYP51 expressed by these plants may be multifunctional, but this had not been confirmed by biochemical characterization previously. The transcriptome of *D. transversa* contained a single *cyp51* candidate transcript (TR348_c1_g1) that was highly expressed in both the leaf (L) and rhizome (R) of the plant (224:236 L:R TPM, Table S1). When translated, this sequence shares 79% ID (90% SIM) and 80% ID (93% SIM) to the *A. thaliana* and *S. lycopersicum* orthologous proteins, respectively (Fig. S5). As such, it was predicted that a single CYP51 (CYP51*Dt*) expressed by *D. transversa* may exhibit substrate promiscuity and function in both the cholesterol (**3**) and phytosterol pathways. In order for P450-mediated catalysis to occur, an NADPH cytochrome P450 reductase (CPR) is required. Generally, for eukaryotic systems, two electrons are funneled from NADPH to the P450 *via* a CPR, permitting activation of molecular oxygen and allowing the oxidation to proceed (16). Having the conspecific CPR usually results in better catalytic activity for a given P450 (17). Thus, analysis of the transcriptome data was undertaken and revealed a single full-length CPR transcript that could be the necessary redox partner for *D. transversa* P450s. This putative reductase (CPR*Dt*) has high homology to both CPR1 (67% ID and 80% SIM) and CPR2 (70% ID and 82% SIM) from *A. thaliana* and contained both the expected FAD- and FMN-binding domains (Fig. S6). In addition, the transcript levels of the putative CPR were high (100:46 L:R TPM, Table S1) in both the rhizome and the leaf, unsurprising given that this protein (CPR*Dt*) appears to be the sole CPR responsible for the delivery of electrons to *D. transversa* P450s.

### Genetic optimization of CYP51*Dt* and CPR*Dt*

The genetic sequences of candidate sterol 14α-demethylase (CYP51*Dt*) and its redox partner (CPR*Dt*) were verified by Sanger sequencing. The coding sequences were then codon optimized for expression in *Escherichia coli*. Native eukaryotic P450s often have a hydrophobic N-terminal region, and heterologous protein expression in *E. coli* is frequently enhanced by replacing this with a shorter and less hydrophobic sequence while retaining the proline-rich hinge region (Pro Pro Ile in CYP51*Dt*) that is important for directing protein folding (17). For *cyp51Dt*, the first 111 base pairs were replaced with a gene sequence encoding MAKKTSSKGKL (18). A C-terminal hexa-histidine tag was introduced to the *cyp51Dt* sequence to facilitate protein purification. The truncated cyp51*Dt* sequence and the full-length *cprDt* sequence were cloned into pCW, a vector that is commonly utilized for P450 expression (19).

### Expression and purification of CYP51*Dt* and CPR*Dt*

**Figure 5:**
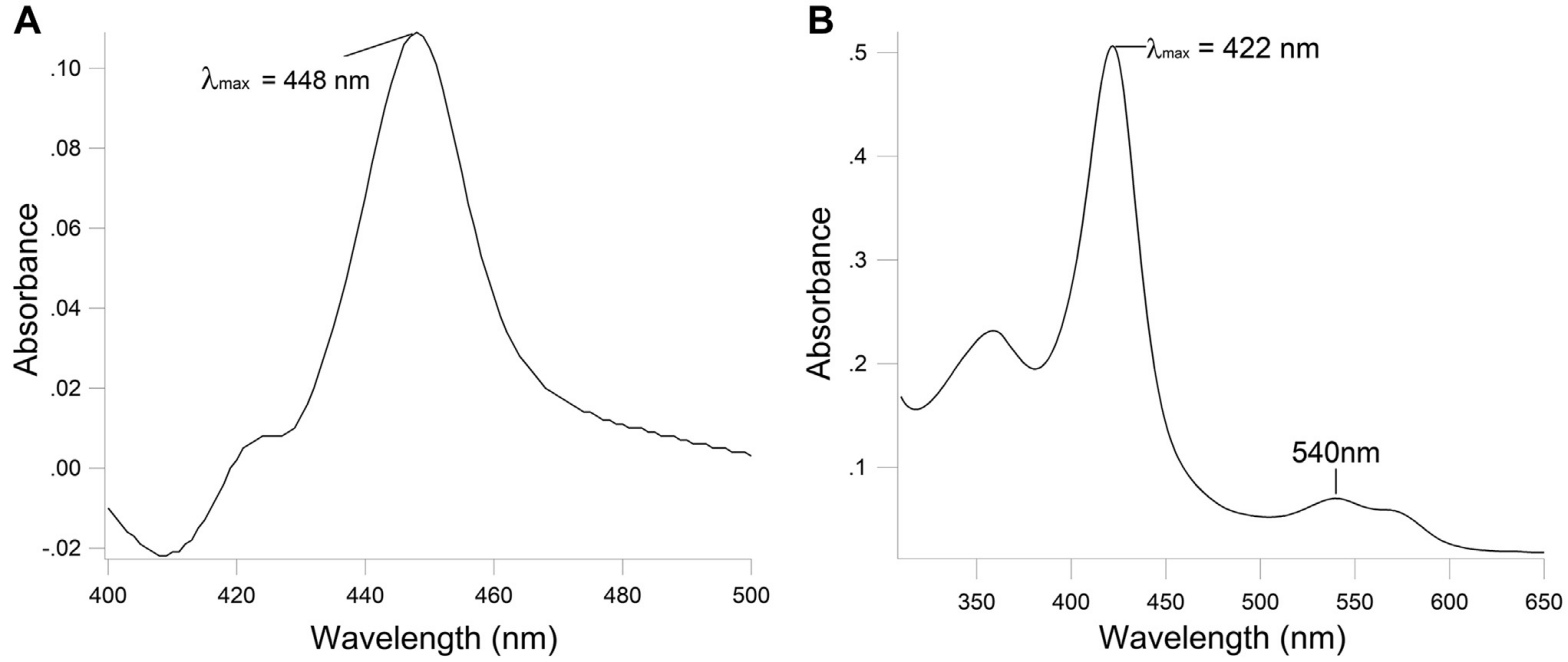
**Spectroscopic Characterization of CYP51*Dt*.***A*, UV–visible difference spectrum of CO complex observed for CYP51*Dt* reduced by sodium dithionite (1.1 μM P450). *B*, UV–visible spectrum of purified CYP51*Dt* (RZ 0.31).

CYP51*Dt* was expressed in *E. coli* from pCW at 25 °C accompanied by the chaperone proteins GroES and GroEL (20). Following successful expression of CYP51*Dt*, purification of the protein was carried out *via* immobilized nickel affinity chromatography and gave 92 nmol P450 L^−1^ of original cell culture. The CO complex of reduced and purified CYP51*Dt* showed a maximum at 448 nm, indicative of properly folded P450 (Fig. 5*A*) (21). The UV–visible spectra of the purified CYP51*Dt* exhibits a Soret band at 422 nm, α/β bands absorbing between 540 and 565 nm, and the δ band at 360 nm, which are all typical features of P450 enzymes (Fig. 5*B*) (22). MS analysis of the trypsin-digested protein identified the protein (65% sequence coverage, Table S2), and SDS-PAGE quantified by densitometry confirmed its purity as >85% (Fig. S7).

The catalytic characterization of CYP51*Dt* required its reductase partner CPR*Dt*. This enzyme was expressed and purified *via* introduction of a hexa-histidine tag to the C terminus. Approximately 50 nmol CPR*Dt* L^−1^ of original expression culture was obtained. The ID of CPR*Dt* was confirmed *via* MS analysis and UV–visible spectroscopy (23).

### CYP51 substrate binding

**Figure 6:**
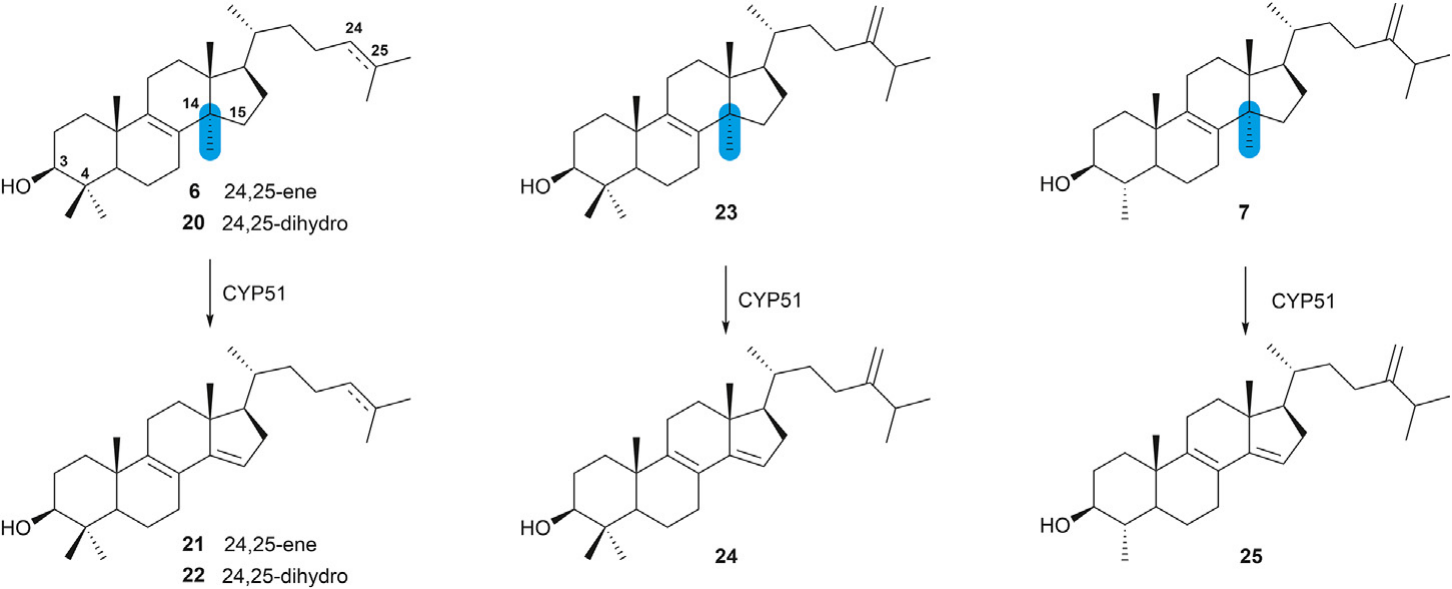
**Theknown demethylation reactions catalyzed by CYP51 enzymes.**

There are four known substrates of CYP51 enzymes (Fig. 6). In animal cholesterol (**3**) biosynthesis, lanosterol (**6**) and 24,25-dihydrolanosterol (**20**) are intermediates that are demethylated by CYP51 to generate **21** and **22**, respectively. Eburicol (**23**) is demethylated by fungal CYP51 enzymes to **24** as part of the ergosterol (**13**) biosynthetic pathway, and plant CYP51s demethylate obtusifoliol (**7**) to **25** in phytosterol metabolism (21).

It was predicted that CYP51*Dt* would accept as substrates obtusifoliol (**7**) and 4-desmethyl-24,25-dihydrolanosterol (**19**) as intermediates for phytosterol and cholesterol (**3**) biosynthesis, respectively. The binding interactions between CYP51*Dt* and **7** and **19** were investigated using UV–visible difference spectroscopy. In a substrate-free P450, the heme ferric iron is low spin (LS Fe^3+^) with a water bound and displays a peak absorbance around 417 nm. This peak shifts to 392 nm characteristic of a high spin iron species (HS Fe^3+^) when a substrate binds and displaces the water bound to the iron in the LS form. The difference spectrum produced from such a binding is referred to as type I spectrum. To explore the substrate specificity of CYP51*Dt*, the LS to HS transition of CYP51*Dt* was also measured in the presence of lanosterol (**6**), 24,25-dihydrolanosterol (**20**), and eburicol (**23**). Our initial binding studies were hampered by typical solubility issues presented by hydrophobic substrates (24). Consequently, we carried out CYP51*Dt*-binding studies in the presence of 5% w/v β-methyl cyclodextrin (BMCD) (25). This can affect apparent *K*_*d*_ values as BMCD competes with the P450 for binding of the sterol (26); however, these values are comparable if measured under the same conditions.

**Figure 7:**
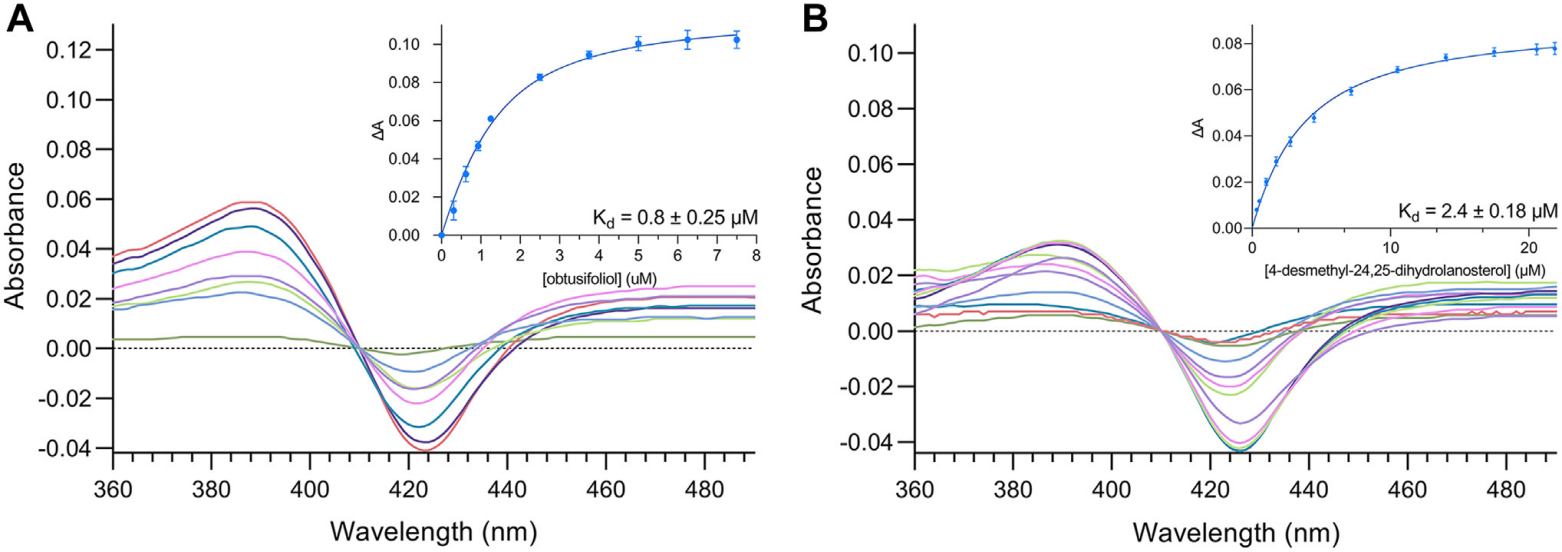
**UV–visible difference spectra and corresponding binding curve ofsubstrate binding topurified CYP51*****Dt*****(1 μM)**. Binding in the presence of increasing concentrations of (*A*) obtusifoliol (**7**) and (*B*) 4-desmethyl-24,25-dihydrolanosterol (**19**) is shown.

Approximately 100% of CYP51*Dt* was converted to HS when obtusifoliol (**7**) was added from an ethanol stock containing 5% w/v BMCD, producing a typical type I P450 spectrum (Fig. 7*A*). The apparent dissociation constant (apparent *K*_*d*_) was determined for purified CYP51*Dt* with obtusifoliol (**7**) and found to be 0.8 ± 0.25 μM, similar to previous reports of eukaryotic P450 affinities, which were also determined in the presence of a cyclodextrin (27). Approximately 80% of CYP51*Dt* was converted to HS by 4-desmethyl-24,25-dihydrolanosterol (**19**), and a typical type I P450 difference spectrum was observed (Fig. 7*B*). The apparent *K*_*d*_ of this interaction was calculated to be 2.4 ± 0.18 μM. It is believed that substrate specificity for C4-monomethyl or 4,4-dimethyl substrates is dependent on whether the enzyme features a phenylalanine (F) or a leucine (L) at a key position in substrate recognition site 1. In cocrystallographic studies, researchers have observed that the C4α-methyl group of obtusifoliol (**7**) resides 4.1 Å below the F, leaving no room for a second methyl group on the β face of the sterol structure (28). CYP51*Dt* possesses an F residue at this position (F109, Fig. S4), and therefore, as expected, titrations of the 4,4-dimethyl sterols **6**, **20**, and **23** with CYP51*Dt* did not produce a type I binding spectrum indicating these are not likely to be good substrates of the enzyme.

### *In vitro* catalytic reconstitution of CYP51

Next, preliminary characterization of the electron transfer from purified CPR*Dt* to CYP51*Dt* was performed *via* CO difference spectroscopy. P450–CO complex formation is only possible after the heme Fe^3+^ is reduced to Fe^2+^ and therefore provides information as to the ability of CPR*Dt* to transfer electrons to the P450. Only 15% of the P450 was reduced by CPR*Dt* in the absence of a steroid, but a much larger proportion was reduced in the presence of a potential substrate (**7**, 100%; **19**, 60%) (Fig. S7). This same phenomenon has been observed previously (29).

**Figure 8:**
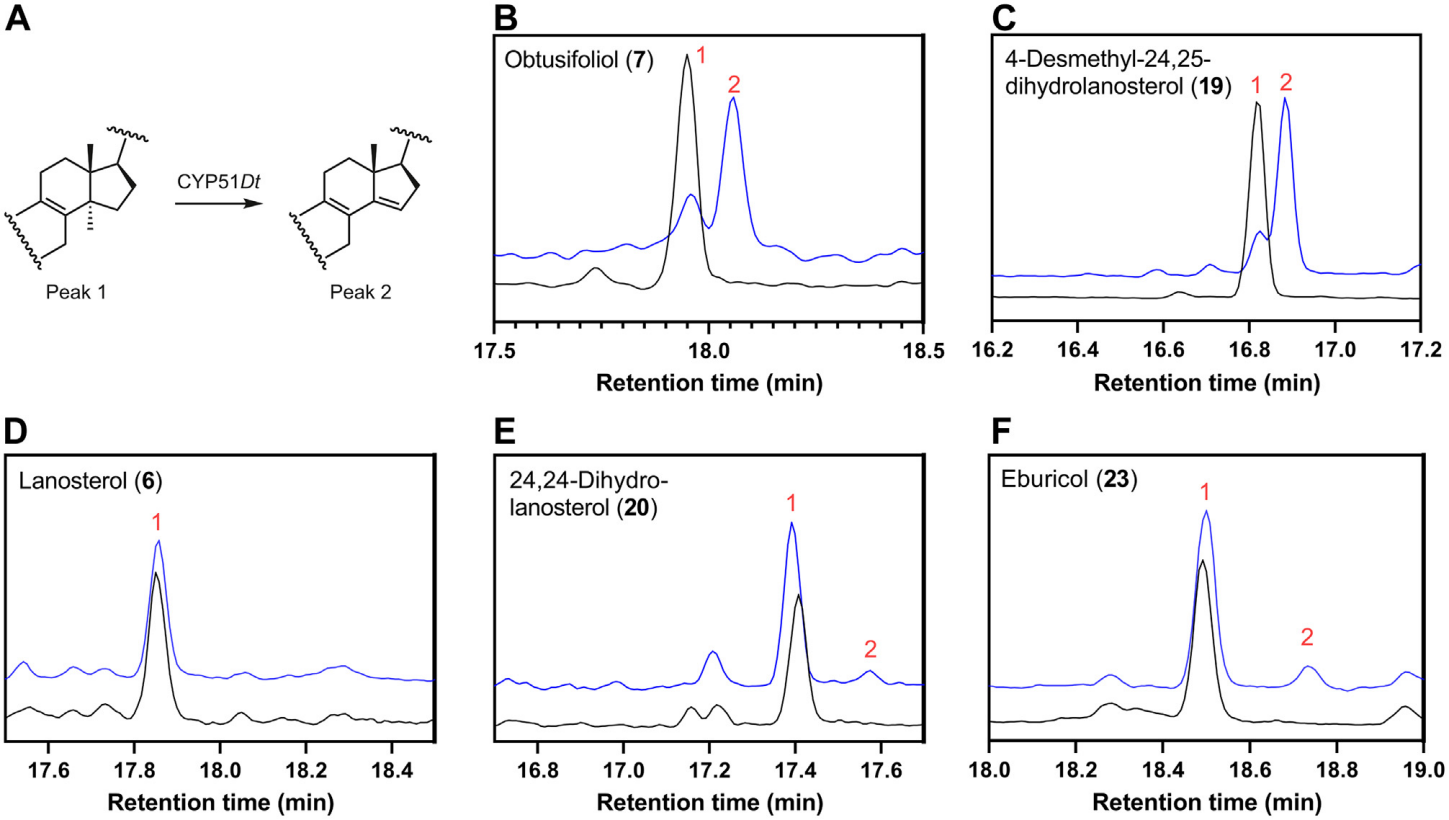
**GC analysis of CYP51*****Dt.**A*, the general reaction performed by CYP51*Dt* and the GC–MS traces of CYP51*Dt* demethylation with substrates (*B*) obtusifoliol (**7**), (*C*) 4-desmethyl-24,25-dihydrolanosterol (**19**), (*D*) lanosterol (**6**), (*E*) 24,25-dihydrolanosterol (**20**), or (*F*) eburicol (**23**). 14α-demethylated products (peak 2) had a slightly longer retention time relative to substrates (peak 1) in all experiments (*blue trace*), which were compared with a control that lacked CPR (*black trace*). All substrates and products were derivatized with BSTFA–TMS before analysis by GC–MS. BSTFA, *N*,*O*-Bis(trimethylsilyl)trifluoroacetamide; CPR, cytochrome P450 reductase; TMS, trimethylsilyl.

**Figure 9:**
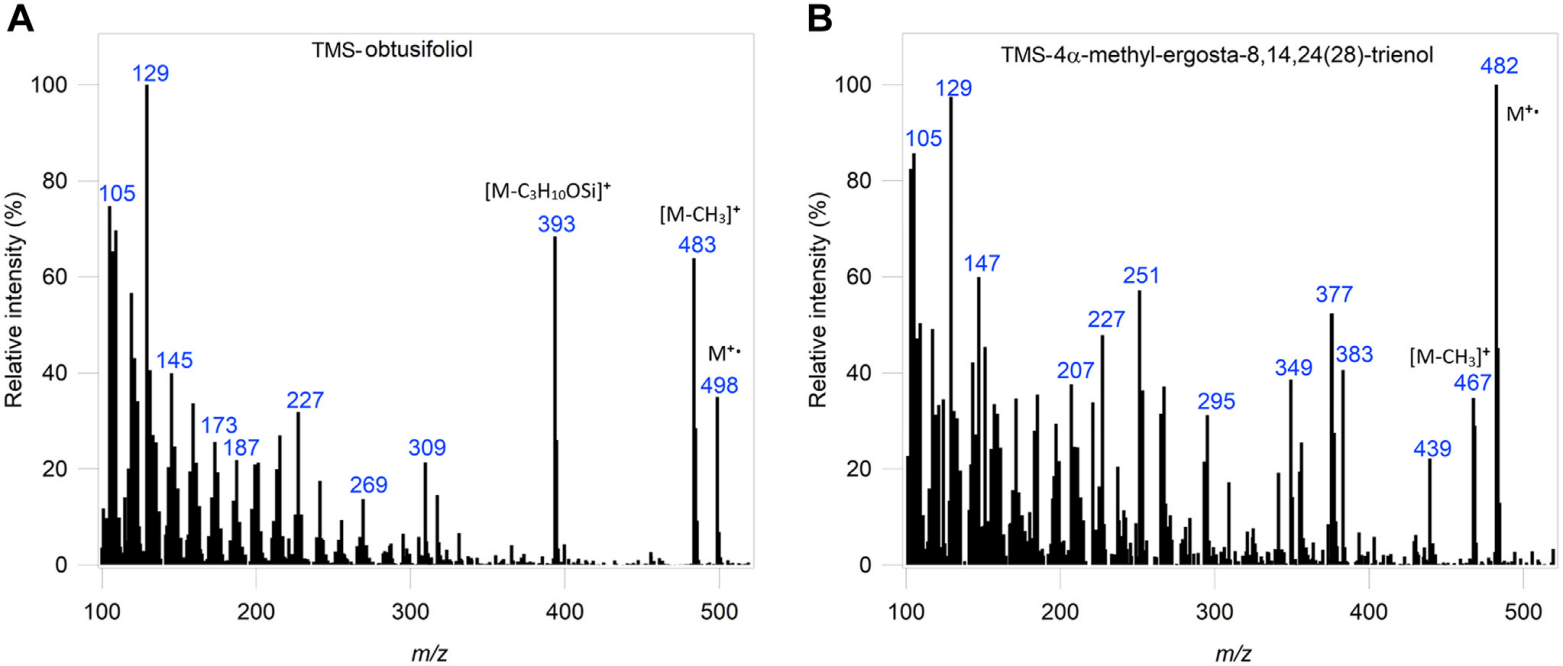
**Mass spectroscopic analysis of CYP51*Dt* catalyzed oxidation of obtusifoliol (7).** Mass spectrum of (*A*) obtusifoliol (**7**) and (*B*) the single new product **25** observed in the turnover of CYP51*Dt* with **7**. Starting material and product were derivatized as the 3-TMS ether before analysis by GC–MS. TMS, trimethylsilyl.

The activity of CYP51*Dt* was then reconstituted *in vitro* with the candidate substrates. Purified CYP51*Dt* was incubated with CPR*Dt* and obtusifoliol (**7**), 4-desmethyl-24,25-dihydrolanosterol (**19**), lanosterol (**6**), 24,25-dihydrolanosterol (**20**), or eburicol (**23**) (Fig. 8). The reactions were initiated by the addition of NADPH, and control reactions lacking either NADPH or CPR*Dt* were run. The organic extracts were analyzed by GC–MS before and after derivatization as trimethylsilyl (TMS) ethers. In CYP51*Dt*-catalyzed oxidation of obtusifoliol (**7**), both analyses showed only one product peak. For example, in the derivatized analysis of **7**, the substrate (retention time = 17.95 min) was largely consumed, and a single new product peak was seen in the GC–MS trace at a retention time of 18.05 min. The MS of the single new product peak possessed a parent ion of the expected mass at *m/z* 482 (M^+•^) when derivatized as the TMS ether (in the underivatized sample, it was at the expected *m/z* 410 [M^+•^]). The mass spectrometric fragmentation pattern (Fig. 9*B*) of the product matched the major peaks of published spectra of both the nonderivatized (30, 31) and TMS-derivatized (32) demethylated product 4α-methylergostatrienol (**25**). This is the expected product from CYP51-mediated demethylation of obtusifoliol (**7**) and a key intermediate in plant production of C24-alkyl phytosterols. In control experiments without CPR, no product formation was observed (Fig. S8).

**Figure 10:**
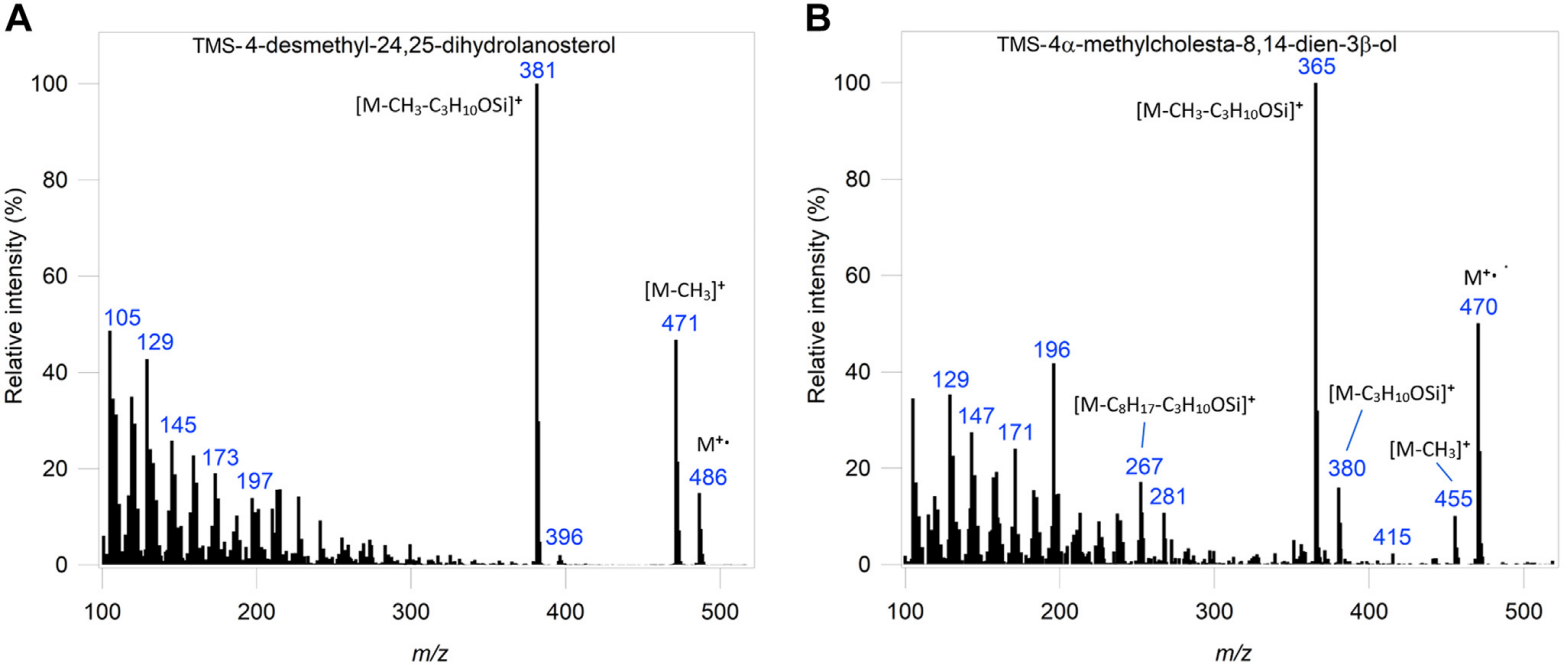
**Mass spectroscopic analysis of CYP51*Dt* catalyzed oxidation of 4-desmethyl-24,25-dihydrolanosterol (19).** Mass spectrum of (*A*) 4-desmethyl-24,25-dihydrolanosterol (19) and (*B*) the single new product 26 observed in the turnover of CYP51*Dt* with 19. Starting material and product were derivatized as the 3-TMS ether before analysis by GC–MS. TMS, trimethylsilyl.

Next, to explore the possibility that CYP51*Dt* is multifunctional, operating in both phytosterol and cholesterol (**3**) biosynthesis in *D. transversa*, we examined CYP51*Dt*-mediated oxidation of 4-desmethyl-24,25-dihydrolanosterol (**19**, Fig. 8*C*). GC–MS analysis of these reactions revealed that essentially complete conversion of **19** (RT = 16.83 min) into a single new product peak at retention time 16.88 min had occurred. The TMS ether of the product possessed a parent ion at *m/z* 470 (M^+•^), and we observed loss of 15 amu (*m/z* 455, M^+•^ −CH_3_^•^) and 105 amu (*m/z* 365, M^+•^ −CH_3_^•^ −C_3_H_10_OSi) (Fig. 10*B*) indicating that the new product and the substrate **19** differ only by a loss of CH_4_ and the introduction of an additional unsaturation. We also observed loss of 203 amu, which correlates with elimination of the TMS (90 amu) and loss of the side chain (113 amu), indicating that the demethylation has occurred on the sterol nucleus (*m/z* 267, M^+•^ −C_3_H_10_OSi −C_8_H_17_^•^). Consequently, the product of this reaction was tentatively identified as the 14α-demethylated product 4α-methyl-cholesta-8,14-dien-3β-ol (**26**). This would be a plausible intermediate in the postulated cholesterol (**3**) biosynthetic pathway, which proceeds from cycloartenol (**4**, Fig. 1).

We also carried out oxidation reactions with the 4,4-dimethyl sterols lanosterol (**6**), 24,25-dihydrolanosterol (**20**), and eburicol (**23**). Despite observing no type I spectra with **23**, catalytic reconstitution with this ligand resulted in a small amount of product observed by GC–MS analysis (Fig. 8*F*). The new TMS-derived product possessed a parent ion at *m/z* 496 (M^+•^) (Fig. S10). The common mass difference between substrate and product (16 amu) once again correlates with elimination of a methyl introducing a double bond. A 281 amu fragment was also observed, which could be associated with elimination of the TMS and loss of the 125 amu side chain (*m/z* 281, M^+•^ −C_9_H_17_^•^ −C_3_H_10_OSi). As such, the new product was tentatively identified as 4,4,24-trimethylcholesta-8,24(28)-dien-3β-ol (**24**). We also observed a small amount of product in turnovers with 24,25-dihydrolanosterol (**20**, Fig. 8*E*). The fragmentation of the product (Fig. S9) and comparison to published spectra (33) supported assignment as the demethylated compound 14-desmethyl-24,25-dihydrolanosterol (**22**). No new product was detected in turnover with lanosterol (**7**, Fig. 8*D*), which differs from **20** only by an additional unsaturation on the sterol side chain. It is possible that CYP51*Dt* catalysis will only occur if C25 is sp^3^ hybridized, which may act as a control mechanism for this promiscuous enzyme.

## Discussion

The steroidal saponins isolated from *D. transversa* are derived from cholesterol (**3**) (1). Preliminary analysis of the *D. transversa* transcriptome suggested that the biosynthesis of **3** itself is highly likely to employ many of the same enzymes used in the biosynthesis of the C24-alkylphytosterols. We propose that in this plant, as has been proposed for others (2, 6), cholesterol (**3**) is derived from cycloartenol (**4**) and that SSR*Dt* initiates cholesterogenesis by reducing cycloartenol (**4**) to cycloartanol (**10**). Within the *D. transversa* transcriptome, homologs of 11 of the 12 enzymes previously proposed (6) for the transformation of 2,3-oxidosqualene to **3** were identified. However, in contrast to previously studied organisms, *D. transversa* appears to not have evolved a unique SSR enzyme for reduction of cycloartenol (**4**) but instead utilizes a single SSR (SSR*Dt*) to act in both phytosterol and cholesterol (**3**) biosynthesis. Through heterologous expression of the *D. transversa* SSR, it was demonstrated that this multifunctional enzyme is capable of reducing both Δ^24,^^28^ double bonds, such as those in phytosterol biosynthesis, and the Δ^24,^^25^ double bond occurring in cycloartenol. The latter reduction would initiate lanosterol-independent cholesterol (**3**) biosynthesis by preventing methylation by an SMT and thus control the biosynthetic flux through the phytosterol biosynthetic pathway relative to cholesterogenesis (2, 6).

Of the 10 enzymes required for the transformation of cycloartanol (**10**) to cholesterol (**3**) and analogously cycloartenol (**4**) to phytosterols, only a single transcript was found for five of them. This implies that they operate in both phytosterol and cholesterol (**3**) biogenesis, as has been postulated previously (6). To investigate this, we determined the substrate specificity and catalytic capability of a key enzyme in steroid biogenesis, CYP51*Dt*. CYP51s are cytochromes P450 responsible for the 14α-demethylation of an intermediate steroid in both phytosterol and cholesterol (**3**) biosynthesis. By performing heterologous expression, purification, and biochemical characterization of the novel *D. transversa* P450 demethylase (CYP51*Dt*), it was demonstrated that the enzyme bound tightly to both obtusifoliol (**7**) and 4-desmethyl-24,25-dihydrolanosterol (**19**). The cognate redox partner of CYP51*Dt* (CPR*Dt*) required for activity was also identified within the transcriptome, heterologously expressed, purified, and characterized.

We confirmed that CPR*Dt* was able to transfer electrons to CYP51*Dt* by observing the formation of the CYP51*Dt*–CO complex, and this was potentiated in the presence of either **7** or **19**. *In vitro* catalytic reconstitution of CYP51*Dt* with CPR*Dt* and NADPH resulted in formation of a single new product from obtusifoliol (**7**), which was identified as 4α-methylergostatrienol (**25**), a known intermediate of phytosterol biosynthesis. In catalytic reconstitutions with 4-desmethyl-24,25-dihydrolanosterol (**19**), a single new product was produced, the molecular ion and fragmentation pattern of which correlated with the expected demethylated product 4α-methylcholesta-8,14-dien-3β-ol (**26**). This compound was previously predicted to be an intermediate of cholesterol (**3**) biosynthesis from cycloartanol (**10**), and thus, it is apparent that the single CYP51 enzyme expressed within *D. transversa* enables biosynthesis of both the C24-alkyl phytosterols and cholesterol (**3**), the precursor of steroidal saponins within the plant (Fig. 1).

**Figure 11:**
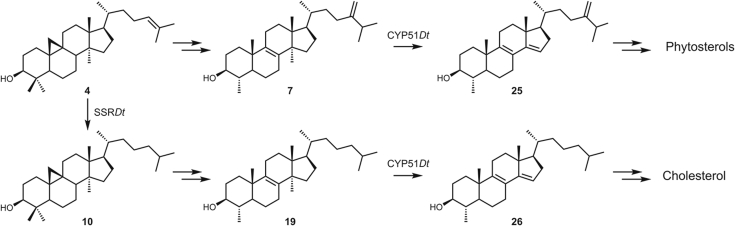
**Overview of the findings in this work.** The SSR present in *D*. *transversa* was shown to be able catalyze the reduction of both a Δ^24,^^25^ and a Δ^24,^^28^ double bond and at high concentrations is postulated to drive the biosynthesis to produce cholesterol instead of phytosterols. CYP51 in this plant was shown to act in both cholesterol and phytosterol biosynthesis, as it was capable of 14α-demethylation of both **7** and **19**. SSR, sterol side-chain reductase.

Consequently, the picture that emerges is one where in *D. transversa*, cholesterol (**3**) is derived from cycloartenol (**4**) and that SSR*Dt* initiates cholesterogenesis by reducing cycloartenol (**4**) to cycloartanol (**10**). Previous studies concluded that *S. tuberosum* (2) and *S. lycopersicum* (6) also generate **3** from **4**, but in contrast to these organisms, *D. transversa* appears not to have evolved a unique SSR enzyme for the reduction of **4**. Instead, it utilizes a single SSR (SSR*Dt*) to act in both phytosterol and cholesterol (**3**) biosynthesis (Fig. 11). The level of expression of SSR*Dt* determines if cycloartenol (**4**) is reduced toward production of cholesterol (**3**) or methylated by an SMT to feed into phytosterol production (*via* **6**). Two parallel pathways then convert cycloartenol (**4**) to phytosterols and cycloartanol (**10**) to cholesterol (**3**, Fig. 1), with several enzymes operating in both routes. SSR*Dt* is capable of Δ^24,^^28^ alkene reduction required at the end of phytosterol biosynthesis. Such substrate promiscuity was also demonstrated for CYP51*Dt* and its associated CPR, which perform the key 14α-demethylation step in both pathways. It remains to be determined if this is a general strategy in plants that produce cholesterol-derived secondary metabolites.

## Experimental procedures

### General experimental

GC–MS spectra were recorded on a Shimadzu GCMS QP2010-plus spectrometer; ZB-5MS column—30 m. Standard GCMS program: column flow 1.7 ml min^−1^; total flow 66.3 ml min^−1^; injector 250 °C; detector 250 °C; oven 200 °C held for 2 min, increased to 300 °C (16 °C min^−1^), and held for 20 min. All chemicals used in this study were analytical grade reagents. UV–visible spectroscopy was performed on a Shimadzu UV-1800 UV–visible spectrophotometer. Commercially available lanosterol (**6**) was converted to 24,25-dihydrolanosterol (**20**) and eburicol (**23**) following literature procedures (34, 35). Obtusifoliol (**7**) and 4-desmethyl-24,25-dihydrolanosterol (**19**) were prepared according to recently developed protocols (36). ^1^H NMR spectra of all substrates are available in Figs. S11–S15.

### Plant material

The *D. transversa* plant material was obtained from the SOWN Nursery, and a specimen was deposited (accession number: PHARM-110003) at the Medicinal Plant Herbarium, Southern Cross University. The plant material was further identified as *Dioscorea* sp. by Dr Hans Wohlmuth (1). The rhizome of the *D. transversa* plant was sliced into 1 to 2 mm sections and snap-frozen in liquid nitrogen in approximately 1 g aliquots. The leaves of *D. transversa* were directly frozen in liquid nitrogen. Both samples were kept at −80 °C until required.

### Steroidal saponin extraction

The steroidal saponins 1β-hydroxyprotoneogracillin (**8**) and protoneogracillin (**9**) were extracted from the *D. transversa* rhizome as outlined in the previously published protocol (1). This was performed to ensure that the plant contained a suitable quantity of steroidal saponins before total RNA extraction. Comparison of HPLC retention times, together with coinjection of standards indicated the presence of the same two steroidal saponins as reported previously. MS was used to confirm the ID of the major saponin, 1β-hydroxyprotoneogracillin (**8**), which gave an *m/z* of 1103.3 under positive ion electrospray ionization conditions (expected [M + Na]^+^ = 1103.5) (1).

### Total RNA preparation and sequencing

The following procedure was modified from a pineapple RNA isolation method developed by Botella *et al.* (37). Total RNA was isolated from either the tuber/rhizome (1.5 g) or the leaf (0.5 g). The frozen plant sample was ground to a powder under liquid nitrogen with a mortar and pestle. RNA extraction buffer (4 ml; 150 mM Tris–HCl [pH 7.5], 2% w/v sodium dodecyl sulphate, 50 mM EDTA, and 1% v/v β-mercaptoethanol) was added, and the sample was mixed for 5 min. Absolute ethanol (0.25 × volume) was added, mixed, and followed by the addition of 5 M potassium acetate (0.11 × volume). One volume of chloroform:isoamylalcohol (24:1) was added to the sample, and the layers were separated by centrifugation (13,000*g*; 3 min). Phenol:chloroform:isoamylalcohol (25:24:1; 1 × volume) was added to the aqueous layer, and again, the layers were separated by centrifugation (13,000*g*; 3 min). Nucleic acids were precipitated by the addition of absolute ethanol (2.5 × volume), which was left for 30 min at −80 °C. The sample was then pelleted (13,000*g*; 30 min) at 4 °C, and the pellet was washed with 80% ethanol before resuspension into RNase-free water (1 ml). The sample was digested with DNase (final concentration of 100 units/μl) for 30 min at RT to remove the contaminating DNA. The DNase was removed by repeating the chloroform:isoamylalcohol and phenol:chloroform:isoamylalcohol steps, and the RNA was precipitated *via* the addition of absolute ethanol (2.5 × volume for 30 min) at −80 °C. The RNA was pelleted (13,000*g*; 30 min; 4 °C), washed with 80% ethanol, and resuspended into RNase-free water (150 μl). LiCl (final concentration of 2 M) was then added to the RNA solution and left overnight at 4 °C to ensure that only RNA remained in the final sample. The following day the RNA was pelleted (13,000*g*; 30 min; 4 °C), washed with 80% ethanol, dried under vacuum, and resuspended into RNase-free water (50 μl). A final RNA concentration step was achieved using the RNA Clean and Concentrator Kit (Zymo Research), and the sample was stored at −80 °C. The total RNA was then submitted to the AGRF for library construction and Illumina HiSeq HT sequencing (100 bp paired-end reads). The two libraries were sequenced on a single lane to produce 113,390,389 paired-end reads for the leaf sample and 116,072,936 paired-end reads for the tuber/rhizome sample.

### Transcriptome construction

#### Read file processing

Initially, SeqyClean (https://github.com/ibest/seqyclean) was used for quality trimming, adapter, poly-A, and contaminant removal for both leaf and tuber/rhizome paired-end files. (k-mer size = 15 and minimum read length = 50). Skewer (https://sourceforge.net/projects/skewer/) (38) was subsequently used to remove adapters that SeqyClean had passed over. For the tuber/rhizome sample, 90.1% of reads were retained, with unpaired reads removed; for the leaf, 95.4% of reads were retained.

#### *De novo* transcriptome assembly

Processed reads from tuber/rhizome and leaf samples were merged and assembled using Trinity (39). The “normalize” option was selected in order to minimize memory requirements. FASTA outputs of Trinity alignments were modified with a custom Python script to minimize header length. TransRate (http://hibberdlab.com/transrate/index.html) (40) was used to generate assembly quality statistics.

#### Functional annotation of generated contigs

To designate a putative function for each contig, multiple approaches were taken. Initially, the longest ORF was extracted from each transcript using Transdecoder (https://transdecoder.github.io/). BLAST searches were conducted locally using BLAST+ against the UniProtKB database (41). Blastp searches were performed with the longest ORF peptides as queries and blastx searches with the contigs as queries. For further characterization of functional domains within translated proteins, both HMMER (http://hmmer.janelia.org/) and PfamScan (http://www.ebi.ac.uk/Tools/pfa/pfamscan/help/) searches were run locally using the Pfam-A database (42). FASTA sequences of contigs annotated as having P450 domains by PfamScan were extracted using custom Python scripts and then imported into the commercial software package Blast2GO (https://www.blast2go.com/) for annotation.

#### Differential expression

Expression was calculated for leaf and tuber/rhizome transcripts using Salmon (https://github.com/COMBINE-lab/Salmon) (43) (TPM) and RSEM (http://deweylab.github.io/RSEM/) (44) (reads per kilobase of transcript per million reads mapped). Differential expression analysis of both transcripts and genes was performed using edgeR46 (45) and DESeq (https://bioconductor.org/packages/release/bioc/) (45) following the suggested Trinity workflow.

### Confirmation of *ssrDt*, *cyp51Dt*, and *cprDt* transcripts

To confirm the genetic sequence of *ssrDt*, *cyp51Dt*, and *cprDt*, cDNA was constructed and used as a template to isolate the ORFs. The cDNA was prepared from total RNA with Superscript III (Invitrogen) following the manufacturer's instructions. Each ORF was then amplified from this cDNA employing specific primers as indicated in Table S3. The following protocol was employed for amplification using a high-fidelity polymerase (Phusion; New England Biolabs): 98 °C for 2 min; 25 cycles of 98 °C (15 s), 50 °C (30 s), and 72 °C (45 s); and 72 °C for 10 min. The resulting DNA product was then used as a template in a second round of amplification employing the same method as outlined previously. This second polymerase reaction allowed the isolation of specific overlapping fragments from each ORF to verify the entire sequence *via* Sanger sequencing (AGRF).

### Generation of mutant BY4741 yeast

Both the pyM44 plasmid and the *S. cerevisiae* strain BY4741 MATa leu2Δ0 met15Δ0 ura3Δ0 erg4Δ0 (*erg4*Δ yeast) were kindly donated by the Professor Benjamin Schulz (University of Queensland). *S. cerevisiae* strain BY4741 MATa leu2Δ0 met15Δ0 ura3Δ0 erg6Δ0 (*erg6*Δ yeast) was generated by subcloning a HISMX gene fragment to disrupt ERG6. The modified cassette HISMX:ERG6 gene fragment was PCR amplified from the bacterial plasmid pyM44 template. The following protocol was employed for amplification using a high-fidelity polymerase (Phusion): 98 °C for 2 min; 25 cycles of 98 °C (15 s), 70 °C (30 s), and 72 °C (45 s); and 72 °C (10 min). The product was extracted from the agarose gel using a QIAquick Gel Extraction Kit (Qiagen) and transformed into WT BY4741 MATa leu2Δ0 met15Δ0 ura3Δ0 yeast (46). Fresh YPAD media (5 ml) were incubated overnight at 30 °C after being inoculated with a single colony of BY4741 yeast. After 16 h, the titer of cells was determined by using a Shimadzu UV-1800 UV–Visible spectrophotometer, and the equivalent of 2.5 × 10^8^ cells was added to a prewarmed flask (30 °C) containing 2× YPAD (50 ml) to yield a final cell titer of 5 × 10^6^ cells ml^−1^. The flask was incubated (30 °C, 200 rpm) until the cell titer reached 2 × 10^7^ cells ml^−1^, at which point the cells were harvested by centrifugation (3000*g*, 5 min), the supernatant was discarded, and the cells were resuspended in sterile ultrapure water (25 ml). The resuspension was centrifuged twice more (3000*g*, 5 min), and each time, the supernatant was discarded and the cells were washed with sterile ultrapure water (1 ml). About 10^8^ cells were resuspended in sterile ultrapure water (100 μl), transferred to a new microcentrifuge tube, and centrifuged once more (13,000*g*, 30 s) The supernatant was discarded, and the cells were resuspended in the transformation mixture (PEG 3350, 50% [w/v], 240 μl; LiAc 1.0 M, 36 μl; denatured salmon sperm 2.0 mg ml^−1^, 50 μl; purified HISxERG6 PCR product, 34 μl) by vortex and incubated (42 °C, 40 min). The mixture was then centrifuged (13,000*g*, 30 s), the supernatant was discarded, and the cell pellet was resuspended in sterile ultrapure water (1 ml) by vortex. The resuspension was plated and grown on yeast nitrogen base containing 2% glucose and synthetic drop-out medium supplements without histidine (Sigma–Aldrich) (72 h, RT). A single colony was then replated onto the same restrictive media to remove background growth. The cells of a single colony were then mixed in an aqueous solution of NaOH (20 mM, 50 μl). DNA extraction was carried out by freeze–thaw method (−78 °C, 2 min; 95 °C, 2 min). Mutation was then confirmed by PCR (Table S4) using a sample of the cells as the template. The following protocol was employed for amplification using a high-fidelity polymerase (Phusion): 98 °C for 2 min; 25 cycles of 98 °C (15 s), 70 °C (30 s) and 72 °C (45 s); and 72 °C (10 min). The mutant colony was then cultured in yeast nitrogen base containing 2% glucose and synthetic drop-out medium supplements without histidine (Sigma–Aldrich) (72 h, 200 rpm). Glycerol stocks of both WT BY4741 and BY4741_M1 were made by mixing culture (500 μl) with an equal volume of glycerol (500 μl) and stored (−80 °C).

### Generation of pRS426GPD.*ssrDt* plasmid and transformation into *erg4*Δ and *erg6*Δ yeast strains

The yeast expression plasmid pRS426GPD was generously donated by Professor Benjamin Schulz. A BamHI site at the 5′-end and an SalI site and the 3′-end was introduced to the confirmed *ssrDt* sequence by PCR (Tables S5 and S6). The synthesized ORF was obtained from General Biosystems as an insert in a pUC57 vector. The gene was excised *via* the BamHI and SalI sites and subsequently ligated into pRS426GPD. Insertion was confirmed by sequencing. BY4741 *erg4*Δ and *erg6*Δ yeast strains were then transformed with the pRS426GPD.*ssrDt* plasmid (46). In this case, transformants were selected by restrictive growth on yeast nitrogen base containing 2% glucose and synthetic drop-out medium supplements without uracil (Sigma–Aldrich).

### GC–MS analysis of cell metabolites

A fresh *erg6*Δ.ssr*Dt* transformant was cultured in YPAD media (5 ml, 30 °C, 200 rpm). Concomitantly, WT BY4741 and BY4741_M1 glycerol stocks were cultured in YPAD media (5 ml, 30 °C, 200 rpm). After 72 h of growth at RT, the cells of each culture were harvested by centrifugation (3000*g*, 5 min) and immediately frozen (−80 °C, 2 h). The frozen cell pellets were placed in a mortar and pestle that had been cooled with liquid nitrogen. The pellets were each crushed to powder in liquid nitrogen and then mixed with 2 M NaOH (0.5 ml) and ethanol (0.5 ml) in order to separate the fatty acids from the other lipids and incubated (47) (80 °C, 1 h). The organic fractions of each mixture were then extracted with hexane (3 × 1 ml), dried over magnesium sulfate, and the solvent evaporated under a stream of nitrogen gas. The dry samples were resuspended in hexane (50 μl) and analyzed by GC–MS. Cell metabolite experiments were performed in triplicate. The ergosterol (**13**) standard was obtained from Sigma–Aldrich.

### Cloning of CYP51*Dt* and CPR*Dt*

Each verified ORF was codon optimized for expression in *E. coli*, and unwanted restriction sites within the sequence were removed. An NdeI site at the 5′-end and a HindIII site at the 3′-end were included in the sequence. For CYP51*Dt*, the first 111 N-terminal base pairs were replaced with a sequence that encodes MAKKTSSKGKL. Both CYP51*Dt*- and CPR*Dt*-synthesized ORFs were obtained from General Biosystems as an insert in the pUC57 plasmid. Each gene was excised from this plasmid and subsequently ligated into the pCW expression vector *via* the NdeI and HindIII sites. To enable purification *via* affinity chromatography, a hexa-histidine tag was introduced to the C termini of both CYP51*Dt* and CPR*Dt via* PCR (Table S7).

### Expression and purification of CYP51*Dt*

Terrific Broth (500 ml) that had been supplemented with thiamine (1 mM), trace element solution (0.025%), ampicillin (100 μg ml^−1^), and chloramphenicol (34 μg ml^−1^) was inoculated with overnight culture (10 ml) of *E. coli* DH5αF’IQ cells transformed with pCW.*cyp51Dt* and the pGro7 plasmid. The culture was incubated for 5 h (25 °C, 150 rpm). IPTG (1 mM) was then added to induce expression of pCW.*cyp51Dt*, and arabinose (4 mg ml^−1^) was added to induce the pGRO7 plasmid. ALA (0.5 mM) was also added to the P450 expression media to promote heme synthesis (48). The culture incubated for 42 h (25 °C, 150 rpm) after which the cells were harvested by centrifugation (4000*g*, 20 min) and immediately frozen (−80 °C). P450 content was calculated according to the protocol of Guengerich *et al*. (*ε* = 91,000 M^−1^ cm^−1^) (49, 50).

All procedures below were performed at 4 °C unless otherwise stated. Purification was carried out on an ÄKTA pure protein purification system (Cytiva). The harvested cells were resuspended in buffer A (100 mM KPi–HCl, pH 7.4, 20% glycerol, 0.5 mM dl-dithiothreitol, 0.1 mM phenylmethylsulfonyl fluoride, 1% CHAPS). Lysis was carried out by sonication on ice (Branson sonifer 450; 3 × 30 s intervals, 30% output), and the cellular debris was removed by centrifugation (20,000*g*, 50 min). The resulting supernatant was filtered (0.45 μm) and diluted 1:1 in buffer B (20 mM KPi, pH 7.4, 500 mM NaCl, 20% glycerol, and 1% CHAPS). This solution was then loaded on to a Ni^2+^-chelating column (5 ml, HiTrap Chelating HP; GE Healthcare) that had been equilibrated with buffer B. The column was washed with five column volumes of buffer B containing 10 mM l-histidine. The column was then washed with an l-histidine gradient in buffer B (10 to 200 mM), and the protein was eluted in 60 mM l-histidine. The protein was dialyzed (17 h × 2) against buffer C (50 mM Tris–HCl, pH 8.0, and 20% glycerol) and immediately frozen (−80 °C).

### Expression and purification of CPR*Dt*

Terrific Broth (500 ml) that had been supplemented with thiamine (1 mM), trace element solution (0.025%), ampicillin (100 μg ml^−1^), and chloramphenicol (34 μg ml^−1^) was inoculated with an overnight culture (10 ml) of *E. coli* DH5αF’IQ cells transformed with pCW.cprDt and the pGro7 plasmid. The culture was incubated (37 °C, 150 rpm) until absorbance at 600 nm reached 0.3, at which point IPTG (1 mM) was added to induce expression of pCW.cprDt and arabinose (4 mg ml^−1^) was added to induce the pGRO7 plasmid. The culture was incubated for a further 42 h (27 °C, 150 rpm), and the cells were harvested by centrifugation (4000*g*, 20 min) and immediately frozen (−80 °C).

All protein purification procedures were performed at 4 °C unless otherwise stated. Purification was carried out on an ÄKTA pure protein purification system (Cytiva). The harvested cells were resuspended in buffer A and stirred on ice for 30 min. Sonication was performed on ice (Branson Sonifer 550, 3 × 20 s intervals, 30% output), and the cellular debris was removed by centrifugation (20,000*g*, 50 min). The resulting supernatant was filtered (0.45 μm) and diluted 1:1 in buffer B and loaded onto a Ni^2+^ chelating column (5 ml, HisTrap Chelating HP; GE Healthcare) that had been equilibrated with buffer B. The column was washed with five volumes of buffer B with 30 mM imidazole, and the protein was eluted with an imidazole gradient in buffer B (30–150 mM). The protein was dialyzed overnight (17 h) against buffer C to remove the imidazole and then immediately frozen (−80 °C). UV–visible spectroscopy of the purified CPR*Dt* showed two peaks at 379 and 453 nm, which is expected for a flavoprotein with an oxidized cofactor (23). SDS-PAGE (Fig. S16) indicated that the CPR was not pure, so fragment MS analysis (later) was used to confirm the presence of CPR*Dt*. The CPR*Dt* yield was estimated by measuring the rate of cytochrome c (*ε* = 110,000 M^−1^ cm^−1^) (49).

### MS of CYP51*Dt* and CPR*Dt*

Purified CYP51*Dt* or CPR*Dt* (56 μg) was loaded onto a 10 kDa molecular weight cutoff centrifugal filter (Merck). The sample was centrifuged (13,000*g*, 10 min), and the flow through was discarded. Ammonium bicarbonate (500 μl, 50 mM) and urea (500 μl, 8 M) were added, the filter was centrifuged (13,000*g*, 20 min), and the supernatant was discarded. The filter was washed again with ammonium bicarbonate (500 μl, 50 mM), and the supernatant was discarded after centrifugation (13,000*g*, 20 min). The sample was resuspended on the filter in ammonium bicarbonate (100 μl, 50 mM) and trypsin (0.5 μg, Promega) and incubated overnight (37 °C, 24 h). The filter was then centrifuged (13,000*g*, 10 min), washed with NaCl (50 μl, 0.5 M), and the filtrate was transferred to a new tube. The filtrate was then subject to a C18 ziptip clean up: a C_18_ ZipTip column (Merck) was washed with buffer 1 (80% acetonitrile, 0.1% formic acid) and equilibrated with buffer 2 (1% acetonitrile, 0.1% formic acid) (3 × 10 μl). The sample was loaded onto the equilibrated column. The column was washed with buffer 1 (3 × 10 μl) and eluted in buffer 2. Solvent was evaporated under vacuum and then resuspended in formic acid (0.1%, 20 μl).

Protein mass spectral analysis of CPR*Dt* and CYP51*Dt* was performed by Dr Amanda Nouwens (University of Queensland). Samples were separated using reversed-phase chromatography on a Dionex Ultimate 3000 RSLC nano-system. Using a flow rate of 30 μl min^−1^, samples were desalted on a Thermo PepMap 100 C18 trap (0.3 × 5 mm, 5 μm) for 3 min, followed by separation on an Acclaim PepMap RSLC C18 (150 mm × 75 μm) column at a flow rate of 300 nl min^−1^. A gradient of 5 to 40% buffer 1 in buffer 2 over 45 min was used to separate the peptides. Eluted peptides were directly analyzed on an Orbitrap Elite mass spectrometer (Thermo) using a nanospray ionization electrospray interface. Source parameters included a capillary temperature of 275 °C; S-Lens RF level at 60%; source voltage of 2 kV; and maximum injection times of 200 ms for MS and 150 ms for MS2. Instrument parameters included an Fourier transform mass spectrometry scan across *m/z* range 350 to 1800 at 60,000 resolution followed by information-dependent acquisition of the top 10 peptides across *m/z* 40 to 1800. Dynamic ion exclusion was employed using a 15 s interval. Charge state screening was enabled with rejection of +1 charged ions, and monoisotopic precursor selection was enabled. Data were converted to mascot generic format using the msConvert software (ProteoWizard) and searched using Protein Pilot, version 5.0 (Sciex). CYP51*Dt* was identified from the search with 65% coverage (10 peptides) within a 95% confidence interval (Table S2). CPR*Dt* was identified with 21.5% sequence coverage (11 peptides) within a 95% confidence interval.

### Apparent dissociation constant (*K*_*d*_) and spin state change

The apparent dissociation constant (*K*_*d*_) of CYP51*Dt* was determined for obtusifoliol (**7**) and 4-desmethyl-24,25-dihydrolanosterol (**19**) by measuring the change in absorbance when the substrate (prepared in an ethanol stock containing BMCD [5% w/v]) was titrated into a solution of purified CYP51*Dt* (1 μM, final substrate concentration: 0.25–40 μM; ethanol concentration did not exceed 1%) using a Shimadzu UV-1800 UV–Visible spectrophotometer. The HS heme content of CYP51*Dt* (1 μM) with a range of steroids was also measured by monitoring the change in absorbance following the addition of the substrate (ethanol stocks with BMCD [5% w/v], final substrate concentration: 100 μM; ethanol concentration did not exceed 1%). Apparent *K*_*d*_ and spin state change experiments were performed in triplicate.

### Catalytic reconstitution of CYP51*Dt* with CPR*Dt*

Purified CPR*Dt* (2 μM) and CYP51*Dt* (1 μM) was added to a solution of Tris–HCl (50 mM, pH 7.4) containing catalase (Sigma–Aldrich) (1 μM) and one of obtusifoliol (**7**), 4-desmethyl-24,25-dihydrolanosterol (**19**), lanosterol (**6**), 24,25-dihydrolanosterol (**20**), and eburicol (**23**) (prepared in an ethanol stock containing 5% w/v BMCD, final substrate concentration of 40 μM). The reaction was initiated with the addition of NADPH (0.4 mM; Sigma–Aldrich) and incubated overnight at RT. The reaction was extracted with ethyl acetate (3 × 2 ml), dried over MgSO_4_, and concentrated under a stream of nitrogen gas. The dried sample was then dissolved in dichloromethane (20 μl), and an equal volume of *N,O*-Bis(TMS)trifluoroacetamide (BSTFA-TMS; Sigma–Aldrich) was added. The sample was heated (water bath at 85 °C, 1 h) before injecting directly onto GC–MS for analysis. Catalytic reconstitution experiments were performed in triplicate.

## Data availability

All data are contained within the article and the supporting information.

## Supporting information

This article contains supporting information.

## Conflict of interest

The authors declare that they have no conflicts of interest with the contents of this article.
