# Supplementary material for: Characterization of the cholesterol biosynthetic pathway in Dioscorea transversa
Source: J Biol Chem. 2023 May 2;299(6):104768. doi: 10.1016/j.jbc.2023.104768 (PMC10267570; doi:10.1016/j.jbc.2023.104768)
Supplement: Supporting information [file mmc1.pdf]

Electronic Supporting Information

Characterization of the cholesterol biosynthetic pathway in *Dioscorea transversa*

Lauren J. Salisbury, Stephen J. Fletcher, Jeanette E. Stok, Luke R. Churchman, Joanne T. Blanchfield and James J. De Voss[a]

[a] School of Chemistry and Molecular Biosciences, The University of Queensland, Brisbane, 4072, Australia.

[a]E-mail: j.devoss@uq.edu.au; Tel: +61 7 3365 3825

Contents

1 Transcripts **1**

2 Verified sequences **1**
2.1 SSR*Dt* 1
2.2 CPR 1
2.3 CPR optimised for expression in *E. coli* 2
2.4 CYP51*Dt* 2
2.5 CYP51*Dt* optimised for expression in *E. coli* 3

3 Mass spectra of yeast metabolites **4**
3.1 *erg4*Δ yeast 4
3.2 *erg4*Δ SSR*Dt* transformant yeast 4
3.3 *erg6*Δ yeast 5
3.4 *erg6*Δ SSR*Dt* transformant yeast 5

4 Supplementary Figures for CYP51*Dt* and CPR*Dt* **6**
4.1 CPR*Dt* sequence alignment 7
4.2 Purified CYP51*Dt* CO-complex 8
4.3 CYP51*Dt* demethylation GCMS analysis 8
4.4 CYP51*Dt* substrate NMR 10
4.5 CYP51*Dt* SDS-PAGE gel 13

1 Transcripts

Table S1 Transcripts in *D. transversa* that were identified as homologues based on their amino acid identity (ID) and similarity (SIM) to known steroid biosynthetic enzymes from *A. thaliana* and the number of present (IF). Transcripts per million (TPM) is a relative measure of expression level used to compare the ratio of expression of a transcript between the leaf (L) and rhizome (R). Enzymes believed to function in both cholesterol and phytosterol biosynthesis in *D. transversa* are highlighted in blue.

Putative enzyme	(L:R) TPM	ID number	ID (SIM)
CAS	38:104	TR2_c1_g1	77(88)
SSR	39:359	TR26180_c2_g1	80(90)
SSR[3]	0.5:0	TR15413_c0_g1	74(87)
CPI	8:17	TR21952_c0_g1	79(87)
SMT1[3]	2.7:0	TR33422_c0_g2	74(88)
SMT1	4.1:1.8	TR37901_c0_g1	72(82)
7-DR1	214:125	TR23093_c0_g1	73(82)
C14-SR	24:22	TR24944_c1_g1	71(84)
C14-SR	46:76	TR24944_c1_g2	73(85)
3βHSD	37:33	TR38268_c0_g1	64(79)
SMO 1	30:0	TR19554_c1_g1	62(77)
SMO 1	43:33	TR19554_c1_g2	69(83)
SMO 1	8.4:0.5	TR11703_c2_g1	63(81)
SMO 2	31:22	TR8738_c3_g3	75(87)
SMO 2 [3]	9.2:3.7	TR8738_c1_g1	77(87)
CYP51	224:236	TR348_c1_g1	79(90)
CPR	190:111	TR12307_c3_g1	70(81)
7,8-SI	127:1.6	TR35422_c0_g1/2	68(80)
7,8-SI	25:6.5	TR23548_c1_g4	69(81)
C5-SD	35:1	TR15783_c0_g1	76(85)
C5-SD	106:29	TR26542_c0_g1	76(85)

2 Verified sequences

2.1 SSR*Dt*

ATGTCGGATCTTCGCACTCCTCTCCGCCCCAAAAGAAAGAAGGTGTTGGTTGATTACCTGGTCCAGTTTCGATGGATAGTTGTTATCTTTGTTGTCCTTCCCATCTCATTC
ACTGTATACTTCTTGTTATATCTTGGCGACATGAGATCTGAAATGAAATCATTCAAGCGTCGCCAAAAGGAACACGATGAGAATGTCAAGAAGGTTGTTAATCGTCTTAAA
CAAAGAGACCCAAAGAAAGATGGACTTGTTTGCACTGCAAGGAAACCATATATTGCCGTTGGCATGCGCAATGTTGATTACAAGCGTGCTAGACATTTTGAGGTTGATCTT
GCGGCATTCAGAAATATTCTTGAGATTGACAAAGAGAGAATGATTGCGAAGGTTGAGCCTCTTGTTAATATGGGCCAGATCAGCAGGGCAACAGTGCCAATGAATCTCTCC
TTAGCTGTTGTTGCAGAACTCGATGATCTTACAGTTGGTGGTCTCATCAATGGCTATGGAATCGAAGGAAGCTCGCATATTTATGGCCTTTTTGCAGACACTGTGGTCGCA
TTGGAACTCGTGCTTGCAGATGGTCGTGTAGTTAGAGCTACAAAGGATAACGAATACTCTGATCTTTTCTATGGTGTGCCCTGGTCACAAGGAACCATTGGGCTTCTAGTT
TCTGCGGAGATCAAGCTTATACCAGTCAAGGAGTATATGAAGTTGACTTACAAGCCGGTGAGGGGGAACTTGAAGGAGCTTGCACAGGCGTATGCTGATTCTTTCGCACCT
AGAGATGGTGACCCAGCGAAAATTCCTGATTTCGTTGAAGCAATGATCTATACTCCGACTGAGGGGGTGATGATGACCGGGAAATATGCTTCCAAGGAGGAAGCCAAGCAA
AAGGGTAATGTCATCAATGAAGTTGGATGGTGGTTTAAAACCTGGTTTTACCAGCATTGCCAGACTGCACTTACAAGGGGTGAGTTCGTGGAATACATTCCCACACGGCAA
TACTACCACAGGCACACGAGGTCCATGTACTGGGAAGGTAAGCTCATTCTGCCATTCGGCGACCAGTGGTGGTTCCGGTGGACCATGGGGTGGTTGATGCCTCCGAAGGTC
TCACTGCTCAAGGCCACTCAGGGTGATGGTATCCGAAACTATTACCATGATATGCATGTGATTCAGGATTTGTTGGTTCCTCTTTACAAAGTCGGTGATTCACTCGAGTTT
GTTCATAGGGAAATGGAGGTGTATCCCATCTGGCTTTGCCCGCATCGCCTCTTCAAGCTCCCTGTGAAAACAATGATTTACCCAGAGCCAGGATTTGAACTACACCGTCGC
CAAGGTGACACAAACAATGCTCAAATGTTCACTGACATTGGTGTGTACTACGCCCCGGGACCTGTCTTGAGGGGCGAAGAATTCAATGGCTCTGAAGCCGTTTGTCGGCTT
GAGGAGTTCTTGATCCAAAACCATGGGTTCCAGCCACAGTACGCGGTCTCTGAACTCACTGAAAAGAACTTCTGGCGAATGTTCGATGGAGAACTCTATGAGAAATGCCGC
CACAAGTATGGTGCCGTAGGTACATTCATGAGTGTGTACTATAAGTCGAAGAAAGGAAAGAAGACTGAGAAGGAGGTGCAAGAGGCCGAGCAGGCGATAGCGGAACAAGCC
TATGCAGAGGATAACTAG

2.2 CPR

ATGAAGCTCTCGGCGCTGGATCTCGTGATGGCGATCCTCACCGGCGGCGATGGGACGGCGCCGGAGGATGCCGCGAGGGAGAGGGTGCTGGCGATCATCGTCACCGCCGGT
TCGCTGCTGATCGGCTGCGTTGTTCTCTTATTTTGGCGCCGGTCGGCCGGCCAGAATAGTAATCGGACGGCGGAGCCGGTGAAGCCTGTCGTCGTCCGGGATGAGGCCGAG

[3]incomplete fragments: SSR, 993 bp fragment; SMT1, 402 bp fragment; SMO2, 513 bp fragment

GATGTGGACGATGGGAAGAAGCGCGTCACTATCTTCTTTGGCACCCAGACCGGCACCGCCGAGGGATTCGCCAAGGCGTTGGCGGAGGAAGCGAGAGCGCGGTATGAAAAG
GCAATATTCAAAATCGTCGATCTGGATGAGTACGCTGCCGATGATGATGAATATGAAGAGAAGATGAAGAAGGAAACTCTTGCCCTGTTCTTCTTAGCAACGTACGGAGAT
GGTGAACCGACTGATAATGCAGCTAGATTCTATAAGTGGTTTACTGAGGGACAAGAGAGGGGAGTTTGGCTTCAAAATCTTCAGTTTGCTGTCTTTGGTCTTGGCAACAGG
CAGTATGAGCATTTCAACAAGGTCGCGAAGGTTGTCGATGAGATTCTTGCTGATCAGGGTGGTAAGCGCCTTGTCCCTGTAGGGCTTGGAGATGATGATCAATGCATTGAG
GATGACTTCAATGCATGGAAAGAGCTTGTATGGCCAGAATTTGATCAATTACTCAGGGATGAAGATGACGTGTCTGGTGCATCAACCCCTTACCAAGCTGCTATACCTGAA
TATCGTATTGTGTTTATTGATTCTGCTGGCGCATCATTCATGGAGAAGAATTGGAATCTTGCCAATGGACATGCTGTTCATGATATCCAACATCCATGCAGAGCTGATGTT
GCTGTAAGGCGAGAACTTCATACACCAGCTTCAGATCGTTCCTGCATTCATTTGGAGTTTGAAATCTCTGGCACTGGATTAGTGTATGAAACAGGAGATCATGTTGGTGTT
TTCTCGGATAATTGTATTGAGACCGTGGAAGAGGCAGAAAGGCTTTTAGGCTATTCATCAGACACATACTTTTCCATTCATGCGAATGAGGGGGATGGCTCACCACGCAGT
GGTGGCTCCTTAGCTCCTCCTTTCCCGTCGCCATGTACTTTGAGAACTGCACTTGCACGATATGCTGACCTTCTGAGTTCACCTAAGAAGGCTGCTTTACTTGCTTTAGCT
GCTCATGCTTCTGAACCCAGTGAGGCTGAACGACTAAGATTTCTAGCTTCCCCTGCTGGGAAGGATGAATATTCTCAATGGATAGTTGCAAGTCAGAGGAGCCTTCTAGAA
GTTATGGCGGAATTTCCATCTGCAAAGCCTCCTCTTGGTGTTTTCTTTGCAGCAATAGCTCCTCGCTTGCAGCCTAGATATTATTCAATATCATCTTCTCCCAAGATGGCA
CCCTCAAGAATTCATGTAACATGTGCCTTGGTCTATGGACCGACACCAACTGGAAGGATTCACAAAGGAGTTTGTTCAACTTGGATGAAGAATGCGACCCCTTGGGATGAA
GATCAGGATTGTAGTTGGGCTCCTGTCTTTGTGAGGCAATCGAATTTTAAACTTCCAGCAGATACAAGTGTACCAATCATCATGATTGGTCCTGGTACAGGGCTAGCTCCT
TTCAGGGGATTCTTGCAGGAACGATTGGCACTGAAGCATTCTGGAGCAGAACTTGGCCAAGCAATTCTCTTCTTTGGATGCAGGAACCGGAAAATTGATTTCATTTATGAG
GATGAATTGAACAATTTTGTTGAGGCAGGTGCACTCTCTGAGCTGATTGTCTCCTTCTCTCGCGAGGGTCCAACCAAGGATTATGTGCAGCATAAGATGACTGAAAAGGCA
TCTGAGTTGTGGCACATCGTCTCCAATGGCGGTTATATTTATGTATGTGGTGATGCTAAGGGGATGGCTAGAGATGTCCATCGCGTTCTTCATACTATAGTCCAAGAGCAA
GGATCCCTGGATAGTTCCAAAGCCGAGAGCATGGTGAAGAGTCTGCAAATGGAAGGGAGATATCTTCGCGATGTTTGG

2.3 CPR optimised for expression in *E. coli*

TTATATCATATGAAACTCAGCGCGCTGGACTTAGTTATGGCAATTCTGACAGGCGGCGACGGCACCGCTCCGGAAGACGCCGCACGCGAACGTGTCCTGGCAATCATCGTG
ACAGCCGGTAGCCTGCTGATTGGTTGTGTGGTGCTGCTGTTCTGGCGTCGCAGCGCAGGCCAGAATTCGAACCGTACCGCCGAGCCCGTTAAACCGGTTGTGGTCCGTGAT
GAAGCTGAAGACGTAGACGACGGGAAAAAAACGCGTCACGATTTTTTTGGGACACAAACAGGGACAGCGGAAGGCTTCGCCAAAGCACTCGCAGAAGAAGCCCGTGCGCGT
TATGAAAAGGCGATTTTCAAGATTGTGGATCTGGATGAATACGCCGCTGATGACGACGAATATGAAGAAAAAATGAAAAAAGAAACTTTGGCATTGTTCTTTCTCGCGACC
TATGGTGATGGCGAACCTACCGATAACGCTGCGCGTTTTTATAAGTGGTTTACGGAAGGTCAAGAACGCGGGGTGTGGCTGCAGAACCTTCAGTTCGCTGTGTTTGGACTG
GGCAATCGTCAATACGAACACTTTAACAAAGTGGCAAAAGTTGTTGACGAAATTTTAGCAGATCAGGGTGGAAAACGTCTGGTCCCGGTTGGCCTGGGCGATGACGACCAG
TGCATCGAGGATGATTTCAATGCCTGGAAAGAGCTCGTATGGCCGGAATTCGATCAGTTGCTGCGTGACGAAGATGACGTCTCCGGTGCGTCCACGCCATACCAGGCAGCG
ATTCCGGAGTACCGCATTGTATTTATTGATTCGGCGGGTGCGAGTTTCATGGAAAAAAACTGGAATCTTGCAAATGGTCACGCCGTCCATGATATCCAACATCCGTGCCGC
GCGGATGTGGCAGTCCGCCGCGAGCTGCACACTCCGGCATCCGATCGTTCCTGTATTCATCTCGAATTCGAAATTAGTGGAACCGGTCTTGTGTATGAAACCGGTGATCAT
GTCGGCGTGTTTTCCGATAACTGCATTGAGACGGTGGAGGAAGCGGAACGTTTACTGGGCTATAGCTCAGACACCTACTTCAGTATTCACGCGAACGAAGGGGATGGCAGC
CCGCGTTCAGGAGGGTCACTTGCACCCCCTTTCCCGTCCCCATGTACCCTGCGTACCGCACTGGCCCGGTACGCAGATCTGCTGAGTAGTCCTAAAAAAGCAGCTCTCCTT
GCGCTGGCCGCACACGCGAGCGAACCCTCCGAAGCAGAGCGCCTGCGTTTTTTGGCCAGCCCGGCTGGTAAAGACGAATACAGTCAATGGATTGTTGCAAGCCAACGCTCA
CTTTTGGAGGTTATGGCGGAATTTCCGAGCGCTAAACCACCTTTAGGCGTGTTTTTCGCCGCGATTGCGCCTCGTCTTCAGCCCCGTTACTACTCCATTTCGAGCAGCCCA
AAAATGGCTCCCTCTCGGATCCACGTAACTTGCGCACTGGTATATGGTCCTACGCCGACCGGCCGCATCCATAAAGGCGTTTGTAGCACATGGATGAAAAATGCGACGCCT
TGGGATGAAGATCAAGATTGTTCATGGGCCCCAGTATTCGTGCGTCAGTCCAACTTTAAACTTCCAGCGGATACATCAGTGCCGATCATTATGATTGGTCCAGGCACAGGA
CTGGCGCCTTTTCGTGGCTTCCTGCAGGAACGTCTTGCTTTGAAACACAGTGGCGCGGAACTTGGCCAGGCGATTTTGTTTTTTGGATGCCGGAACCGTAAAATTGACTTT
ATTTACGAAGATGAACTGAATAATTTTGTTGAAGCTGGCGCTCTGTCTGAGTTGATTGTGAGTTTTTCACGCGAAGGCCCGACCAAAGATTATGTTCAGCACAAAATGACA
GAAAAAGCGTCGGAGCTTTGGCACATCGTAAGTAACGGGGGATACATCTATGTGTGTGGCGACGCAAAAGGTATGGCACGCGATGTTCATCGCGTACTTCATACTATTGTG
CAGGAGCAAGGCAGTCTGGATTCTAGCAAAGCCGAATCAATGGTTAAATCCCTGCAAATGGAAGGCCGGTATTTGCGTGATGTTTGGTAGGGTACCAAGCTTTTATAT

2.4 CYP51*Dt*

ATGGATCTCAAGGAGAACAAGTTCTTCAGCATTGGCCTTGTCTTCCTGGCCACTATCATCTTCATCAAGCTCTTGGCTGCGCTTTTCCGGTCCGGATCCAAGCACCGGAAG
CCGCCGATCGTCAAATCGTTGCCGGTGATCGGAGGGCTTCTCCGATTCATGAAAGGCCCCATCTTGCTGATCCGCCAGGAGTATCAGAAGCTTGGGAGCGTGTTCACGCTC
AACATTGTAAACCGAAAGATCACCTTTTTCGTTGGTCCCGAGGTGTCCTCGCACTTCTTCAAGGCGCCTGAGGTGGAGCTCAGTCAGCAGGAGGTGTACCAGTTCAATGTA
CCCACCTTCGGCCCTGGGGTTGTTTTCGATGTGGATTACTCTGTGCGGCAGGAGCAGTTCCGCTTCTTCACGGAATCGCTTAGAGTGAGCAAGCTCAAGAGCTATGTCGAC
CAGATGGTTGTGGAGGCTGAGGATTACTTCTCAAAGTGGGGAGAGTGTGGTGAAGTGGATCTAAAATATGAGCTGGAACATCTTATCATTCTAACGGCAAGCAGGTGCCTT
CTTGGCAGGGAGGTGAGAGACAAACTCTTTGATGATGTCTCCGCGCTCTTCCATGACCTTGACAATGGCATGATCCCCATTAGCGTCATCTTCCCGTACCTCCCAATCCCT
GCTCACCGCCGTCGTGATAGGGCCCGAGCAAGAATTGCTGAGATCTTTTCAACCATTATCAATTCCCGCAAGGTGTCTGGCAAGTCTGAGGATGACATGCTGCAGTGTTTT
ATTGAGTCCAAGTACAAGGATGGCCGCCCAACCACTGACGGTGAAATTACTGGGCTTCTCATTGCTGCACTCTTTGCTGGCCAACACACCAGCTCCATTACATCCACCTGG
ACTGGTGCCTACCTGCTCCGCTTCAAGCAATACCTCGCAGCTGCACTTGATGAACAGAAGCAGCTTATGAAAAAGCACGGGGATAAGGTCAACCATGATATTTTAGCAGAG
ATGGATGTGCTCTATCGCTGCATCAAGGAGGCTCTGAGACTCCACCCACCTCTAATAATGCTGCTCCGTCAATCTCATTCTGACTTCACTGTGAAAACCAAGGAAGGGATT
GAGTATGATATCCCAAAGGGCCACATTGTTGCCACTTCTCCTGCTTTTGCAAACCGACTTCCTTACATCTACAAGGATCCTGATACATATGACCCAGACCGTTTTGTCCCT
GGTAGAGAGGAGGATAAGGTTGCCGGGGCCTTCTCATACATTTCATTCGGGGGTGGCAGGCACGGGTGCCTTGGTGAACCATTTGCTTACTTGCAGATAAAGGCGATCTGG
ACTCATTTGTTGAGGAACTTTGAATTCGAGCTTGTCTCTCCCTTCCCTGAGATTGATTGGAATGCCATGGTGGTTGGTGTGAAAGGGCAGGTGATGGTGAGGTACAAGCGG
AGGAAGCTGTCTGTTGATAGCTGA

2.5 CYP51*Dt* optimised for expression in *E. coli*

TTATATCATATGGACTTAAAAGAGAACAAGTTTTTTTCAATCGGTTTGGTGTTTCTGGCGACGATTATCTTTATAAAATTATTGGCCGCACTGTTCAGAAGCGGGAGTAAG
CACCGGAAACCACCTATCGTCAAATCACTTCCTGTTATCGGTGGGCTGCTTAGATTTATGAAAGGACCAATACTGTTAATCCGGCAAGAATATCAGAAGTTAGGCAGTGTT
TTTACATTGAACATAGTGAACAGAAAGATAACCTTTTTTGTTGGTCCCGAGGTGTCTAGCCATTTTTTCAAGGCTCCAGAAGTGGAGTTATCTCAACAGGAAGTCTACCAG
TTCAATGTGCCCACCTTTGGCCCTGGCGTGGTTTTTGACGTCGACTATAGTGTACGGCAGGAGCAGTTTCGTTTTTTTACAGAGTCACTGCGCGTCAGTAAGTTAAAATCC
TATGTTGATCAAATGGTCGTTGAAGCTGAAGACTATTTCAGTAAATGGGGTGAATGCGGGGAAGTGGACTTGAAGTACGAACTGGAACACTTGATTATCTTAACCGCTAGT
AGATGTTTACTGGGGCGCGAGGTCCGCGACAAACTGTTTGATGATGTCAGTGCCCTGTTTCATGACCTTGATAATGGGATGATTCCAATATCTGTGATTTTCCCGTATTTA
CCAATCCCCGCACATCGCCGTCGGGACAGAGCCAGAGCGAGAATAGCAGAGATTTTTTCCACCATTATTAATTCACGTAAGGTCTCGGGGAAGTCAGAAGATGACATGCTG
CAATGTTTTATCGAGTCTAAGTACAAGGACGGGCGCCCTACAACAGACGGCGAAATCACTGGATTGCTGATAGCGGCTCTGTTTGCTGGACAGCATACCTCGTCGATTACT
TCTACATGGACAGGCGCTTATCTTCTGCGGTTCAAGCAATACCTGGCCGCCGCGCTGGACGAGCAAAAGCAGCTTATGAAGAAACACGGCGATAAAGTTAATCATGACATA
TTAGCCGAGATGGATGTGCTGTATCGCTGTATAAAAGAGGCGTTAAGACTGCATCCTCCTCTTATCATGTTGTTGAGACAATCTCATTCAGATTTCACCGTCAAAACAAAA
GAAGGCATCGAGTACGATATACCCAAAGGTCACATTGTCGCAACGAGTCCTGCATTCGCAAACCGTTTACCTTACATTTATAAAGATCCGGATACTTACGACCCGGACAGA
TTCGTACCGGGCAGAGAGGAGGATAAAGTCGCCGGCGCATTCTCATATATTTCTTTCGGTGGTGGGCGCCATGGATGCTTGGGGGAGCCGTTCGCTTATCTTCAAATTAAG
GCGATATGGACTCATCTTCTTCGGAACTTTGAGTTCGAACTTGTGTCTCCCTTCCCCGAGATAGACTGGAACGCTATGGTTGTAGGCGTTAAAGGGCAGGTAATGGTGCGC
TATAAACGTCGGAAGCTGAGTGTTGATAGTTAGGGTACCAAGCTTTTATAT

3 Mass spectra of yeast metabolites

3.1 *erg4*Δ yeast

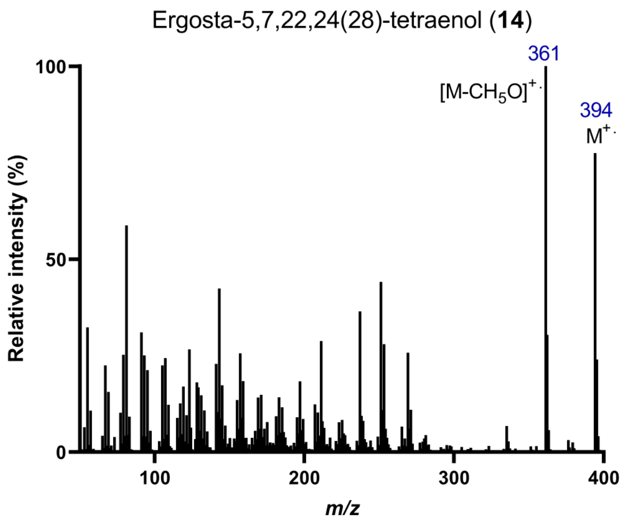

Figure S1 The fragmentation patterns of ergosta-5,7,22,24(28)-tetraenol (**14**) in Figure 3a.

3.2 *erg4*Δ SSR*Dt* transformant yeast

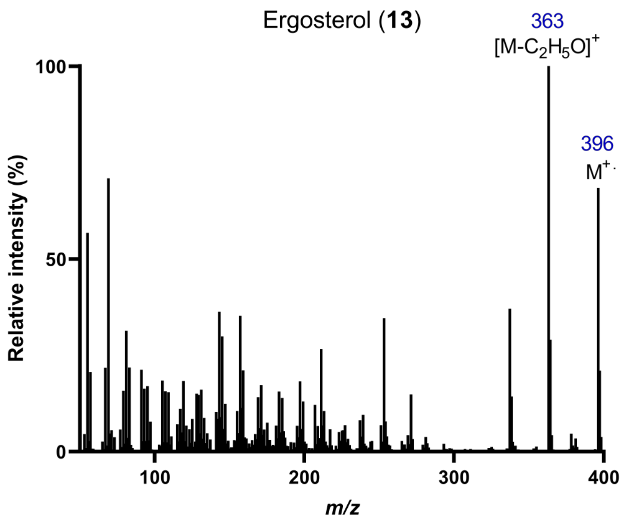

Figure S2 The fragmentation patterns of ergosterol (**13**) in Figure 3a and 3b.

3.3 *erg6*Δ yeast

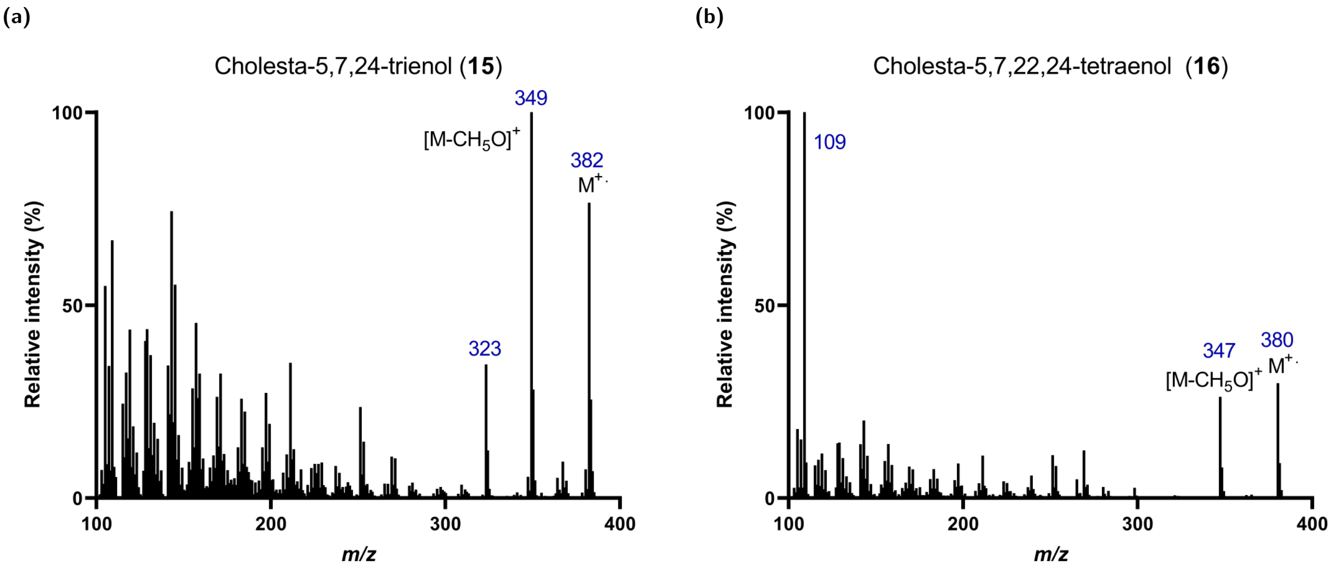

Figure S3 The fragmentation patterns of (a) cholesta-5,7,24-trienol (**15**) and cholesta-5,7,22,24-tetraenol (**16**) in Figure 3b.

3.4 *erg6*Δ SSR*Dt* transformant yeast

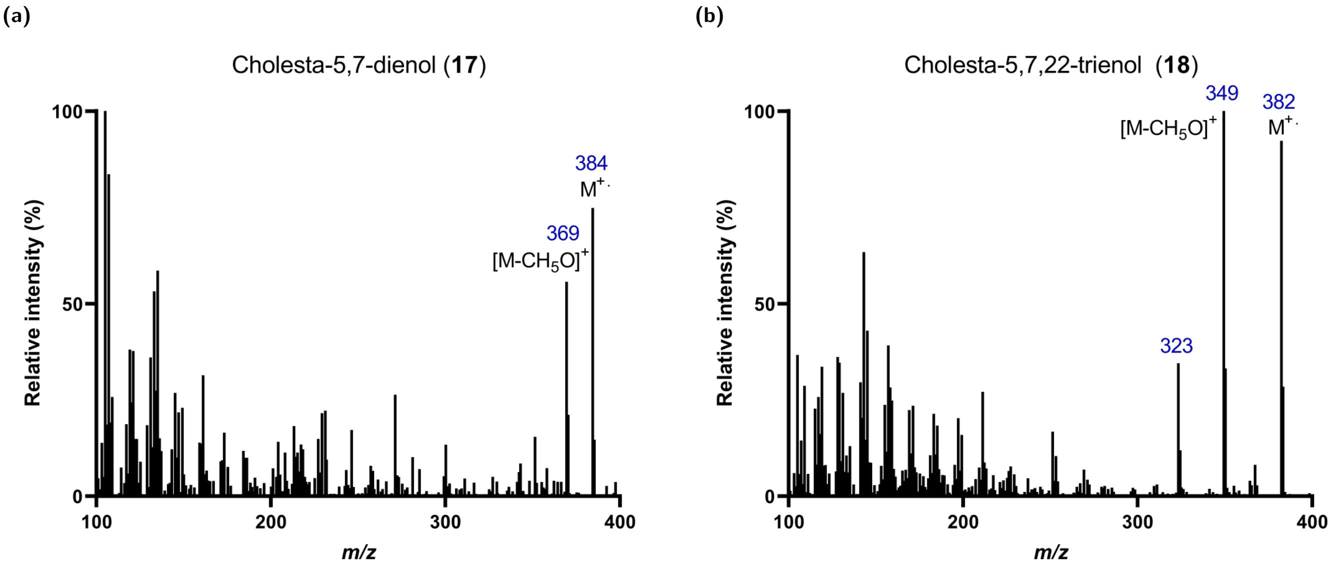

Figure S4 The fragmentation patterns of (a) cholesta-5,7-dienol (**17**) and cholesta-5,7,22-trienol (**18**) in Figure 3b.

4 Supplementary Figures for CYP51*Dt* and CPR*Dt*

subsectionCYP51*Dt* amino acid sequence alignment

```
D. transversa      1 MDLK-ENKFFSIGLVFLATIIFIKLLAALFRSGSK-HRKPPIVKSLP-VIGGLLRFMKGP
S. lycopersicum    1 MELG-DNKILNVGLLLVATLLVAKLISALIMPRSK-KRLPPVVKAWP-IVGGLIRFLKGP
A. thaliana        1 MELDSENKLLKTGLVIVATLVIAKLIFSFFTSDSKKKRLPPTLKAWPPLVGSLIKFLKGP
                                                                      SRS1
D. transversa     58 ILLIRQEYQKLGSVFTLNIVNRKITFFVGPEVSSHFFKAPEVELSQQEVYQFNVPTFGPG
S. lycopersicum   58 VVMLRQEYPKLGSVFTLNLLNKNITFFIGPEVSAHFFKAPETDLSQQEVYQFNVPTFGPG
A. thaliana       61 IIMLREEYPKLGSVFTVNLVHKKITFLIGPEVSAHFFKASESDLSQQEVYQFNVPTFGPG

D. transversa    118 VVFDVDYSVRQEQFRFFTESLRVSKLKSYVDQMVVEAEDYFSKWGECGEVDLKYELEHLI
S. lycopersicum  118 VVFDVDYTIRQEQFRFFTEALRVTKLKGYVDQMVTEAEEYFSKWGESGEVDLKYELEHLI
A. thaliana      121 VVFDVDYSVRQEQFRFFTEALRVNKLKGYVDMMVTEAEDYFSKWGESGEVDIKVELERLI
                                                SRS2                SRS3
D. transversa    178 ILTASRCLLGREVRDKLFDDVSALFHDLDNGMIPISVIFPYLPIPAHRRRDRARARIAEI
S. lycopersicum  178 ILTASRCLLGEEVRNKLFDDVSALFHDLDNGMLPISVIFPYLPIPAHRRRDNARKKLAEI
A. thaliana      181 ILTASRCLLGREVRDQLFDDVSALFHDLDNGMLPISVLFPYLPIPAHRRRDRAREKLSEI
                                                                     SRS4
D. transversa    238 FSTIINSRKVSGKSEDDMLQCFIESKYKDGRPTTDGEITGLLIAALFAGQHTSSITSTWT
S. lycopersicum  238 FANIINSRKRTGKAENDMLQCFIDSKYKDGRPTTEGEITGLLIAALFAGQHTSSITSTWT
A. thaliana      241 FAKIIGSRKRSGKTENDMLQCFIESKYKDGRQTTESEVTGLLIAALFAGQHTSSITSTWT

D. transversa    298 GAYLLRFKQYLAAALDEQKQLMKKHGDKVNHDILAEMDVLYRCIKEALRLHPPLIMLLRQ
S. lycopersicum  298 GSYLLTNDKYMSAVVDEQKNLMKKHGNKVDHDILSEMEVLYRCIKEALRLHPPLIMLLRS
A. thaliana      301 GAYLMRYKEYFSAALDEQKNLIAKHGDKIDHDILSEMDVLYRCIKEALRLHPPLIMLMRA
                                                SRS5
D. transversa    358 SHSDFTVKTKEGIEYDIPKGHIVATSPAFANRLPYIYKDPDTYDPDRFVPGREEDKVAGA
S. lycopersicum  358 SHSEFSVTTREGKEYDISKGHIVATSPAFANRLPHIFKNPDTYDPDRFGPGREEDKAAGA
A. thaliana      361 SHSDFSVTARDGKTYDIPKGHIVATSPAFANRLPHIFKDPDTYDPERFSPGREEDKAAGA

D. transversa    418 FSYISFGGGRHGCLGEPFAYLQIKAIWTHLLRNFEFELVSPFPEIDWNAMVVGVKGQVMV
S. lycopersicum  418 FSYISFGGGRHGCLGEPFAYLQIKAIWSHLLRNFEFELISPFPEIDWNAMVVGVKGEVMV
A. thaliana      421 FSYIAFGGGRHGCLGEPFAYLQIKAIWSHLLRNFELELVSPFPEIDWNAMVVGVKGNVMV

D. transversa    478 RYKRRKLSVDS
S. lycopersicum  478 KYKRRKLSVE-
A. thaliana      481 RYKRRQLS---
```

Figure S5 Amino acid sequence alignment of CYP51*Dt* from *D. transversa* (TR12307_c3_g1) with CYP51 from *S. lycopersicum* (D9J0A9_SOLLC) and *A. thaliana* (CP511_ARATH). Substrate recognition sites highlighted in red and key residue **F109** is bolded. Amino acid sequences were aligned using the software T. Coffee and graphic was generated using pyBoxshade.

4.1 CPR*Dt* sequence alignment

```
CPR1     1 ---------------MTS----------ALYASDLFKQLKSIMGTDSLSDDVVLVIATTSL
CPRDt    1 ------MKLSALDLVMAIILTGGDGTAPED-----------------AA-RERVLAIIVTAG
CPR2     1 MSSSSSSSTSMIDLMAAIIKGEPVIVSDPANASAYESVAAELSSMLIENRQFAMIVTTSI

CPR1    37 AL-VAGFVVLLWKKTTADRSGELKPLMIPKSLMAKDEDDDLDLGSGKTRVSIFFGTQTGT
CPRDt   38 SLLIGCVVLLFWRRSAGQNSNRTAEPVK--PVVVRDE--AEDVDDGKKRVTIFFGTQTGT
CPR2    61 AVLIGCIVMLVWRRSGSGNS-KRVEPLK--PLVIKPR--EEEIDDGRKKVTIFFGTQTGT

CPR1    96 AEGFAKALSEEIKARYEKAAVKVIDLDDYAADDDQYEEKLKKETLAFFCVATYGDGEPTD
CPRDt   94 AEGFAKALAEEARARYEKAIFKIVDLDEYAADDDEYEEKMKKETLALFFLATYGDGEPTD
CPR2   116 AEGFAKALGEEAKARYEKTRFKIVDLDDYAADDDEYEEKLKKEDVAFFFLATYGDGEPTD

CPR1   156 NAARFSKWFTEENERDIKLQQLAYGVFALGNRQYEHFNKIGIVLDEELCKKGAKRLIEVG
CPRDt  154 NAARFYKWFTEGQERGVWLQNLQFAVFGLGNRQYEHFNKVAKVVDEILADQGGKRLVPVG
CPR2   176 NAARFYKWFTEGNDRGEWLKNLKYGVFGLGNRQYEHFNKVAKVVDDILVEQGAQRLVQVG

CPR1   216 LGDDDQSIEDDFNAWKESLWSELDKLLKDEDDK-SVATPYTAVIPEYRVVTHDPRFTTQK
CPRDt  214 LGDDDQCIEDDFNAWKELVWPEFDQLLRDEDDVSGASTPYQAAIPEYRIVFIDSAGASFM
CPR2   236 LGDDDQCIEDDFTAWREALWPELDTILREEGDT-AVATPYTAAVLEYRVSIHDSEDAKFN

CPR1   275 SMESNVANGNTTIDIHHPCRVDVAVQKELHTHESDRSCIHLEFDISRTGITYETGDHVGV
CPRDt  274 EKNWNLANGHAVHDIQHPCRADVAVRRELHTPASDRSCIHLEFEISGTGLVYETGDHVGV
CPR2   295 DITLANGNGYTVFDAQHPYKANVAVKRELHTPESDRSCIHLEFDIAGSGLTMKLGDHVGV

CPR1   335 YAENHVEIVEEAGKLLGHSLDLVFSIHADKEDGSPLES-AVPPPFPGPCTLGTGLARYAD
CPRDt  334 FSDNCIETVEEAERLLGYSSDTYFSIHANEGDGSPRSGGSLAPPFPSPCTLRTALARYAD
CPR2   355 LCDNLSETVDEALRLLDMSPDTYFSLHAEKEDGTPISS-SLPPPF-PPCNLRTALTRYAC

CPR1   394 LLNPPRKSALVALAAYATEPSEAEKLKHLTSPDGKDEYSQWIVASQRSLLEVMAAFPSAK
CPRDt  394 LLSSPKKAALLALAAHASEPSEAERLRFLASPAGKDEYSQWIVASQRSLLEVMAEFPSAK
CPR2   413 LLSSPKKSALVALAAHASDPTEAERLKHLASPAGKDEYSKWVVESQRSLLEVMAEFPSAK

CPR1   454 PPLGVFFAAIAPRLQPRYYSISSCQDWAPSRVHVTSALVYGPTPTGRIHKGVCSTWMKNA
CPRDt  454 PPLGVFFAAIAPRLQPRYYSISSSPKMAPSRIHVTCALVYGPTPTGRIHKGVCSTWMKNA
CPR2   473 PPLGVFFAGVAPRLQPRFYSISSSPKIAETRIHVTCALVYEKMPTGRIHKGVCSTWMKNA

CPR1   514 VPAEKSHEC-SGAPIFIRASNFKLPSNPSTPIVMVGPGTGLAPFRGFLQERMALKEDGEE
CPRDt  514 TPWDEDQDC-SWAPVFVRQSNFKLPADTSVPIIMIGPGTGLAPFRGFLQERLALKHSGAE
CPR2   533 VPYEKSEKLFLGRPIFVRQSNFKLPSDSKVPIIMIGPGTGLAPFRGFLQERLALVESGVE

CPR1   573 LGSSLLFFGCRNRQMDFIYEDELNNFVDQGVISELIMAFSREGAQKEYVQHKMMEKAAQV
CPRDt  573 LGQAILFFGCRNRKIDFIYEDELNNFVEAGALSELIVSFSREGPTKDYVQHKMTEKASEL
CPR2   593 LGPSVLFFGCRNRRMDFIYEEELQRFVESGALAELSVAFSREGPTKEYVQHKMMDKASDI

CPR1   633 WDLIKEEGYLYVCGDAKGMARDVHRTLHTIVQEQEGVSSSEAEAIVKKLQTEGRYLRDVW
CPRDt  633 WHIVSNGGYIYVCGDAKGMARDVHRVLHTIVQEQGSLDSSKAESMVKSLQMEGRYLRDVW
CPR2   653 WNMISQGAYLYVCGDAKGMARDVHRSLHTIAQEQGSMDSTKAEGFVKNLQTSGRYLRDVW
```

Figure S6 Sequence alignment of CPR*Dt* from *D. transversa*, with *A. thaliana* CPR1 (X66016) and CPR2 (X66017). The residues of the FMN binding domain are indicated in red; and the residues of the FAD binding domain are shown in blue.Amino acid sequences were aligned using the software T. Coffee and graphic was generated using pyBoxshade.

4.2 Purified CYP51*Dt* CO-complex

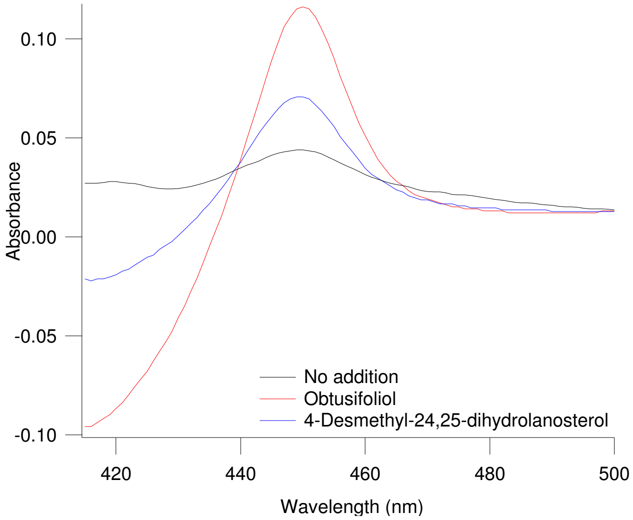

Figure S7 (a) CO-complex observed of purified CYP51*Dt* (1 μM) incubated with purified CPR*Dt* (1 μM) in the presence of NADPH (0.2 mM) in the presence of obtusifoliol (10 μM) or with 4-desmethyl-24,25-dihydrolanosterol (10 μM) (both substrates were dissolved in ethanol with 5% *w/v* BMCD; ethanol concentration did not exceed 1%).

4.3 CYP51*Dt* demethylation GCMS analysis

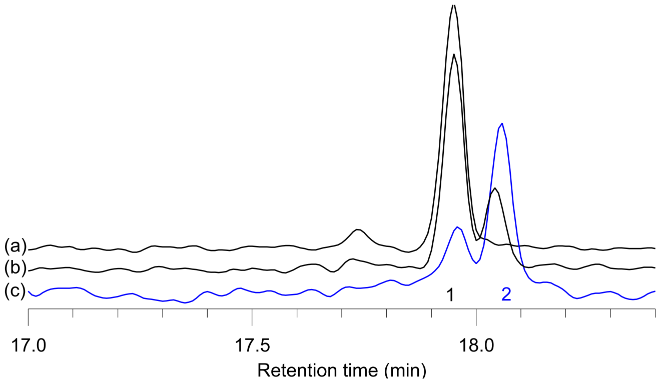

Figure S8 GCMS trace comparing the products from CYP51*Dt* turnover of obtusifoliol (c), the -CPR control (a) and the -NADPH control (b). Obtusifoliol (peak 1) eluted at 17.95 min and the new product eluted at 18.05 min (peak 2). All products and standards were derivatised with BSTFA-TMS before analysis by GCMS. Interestingly, a small amount of product was produced in the control reaction (b) which lacked only NADPH, but this product was absent in the -CPR*Dt* control. This could indicate that some of the purified CPR*Dt* was already in its reduced form prior to the addition of NADPH, and thus was able to provide electrons to CYP51*Dt*

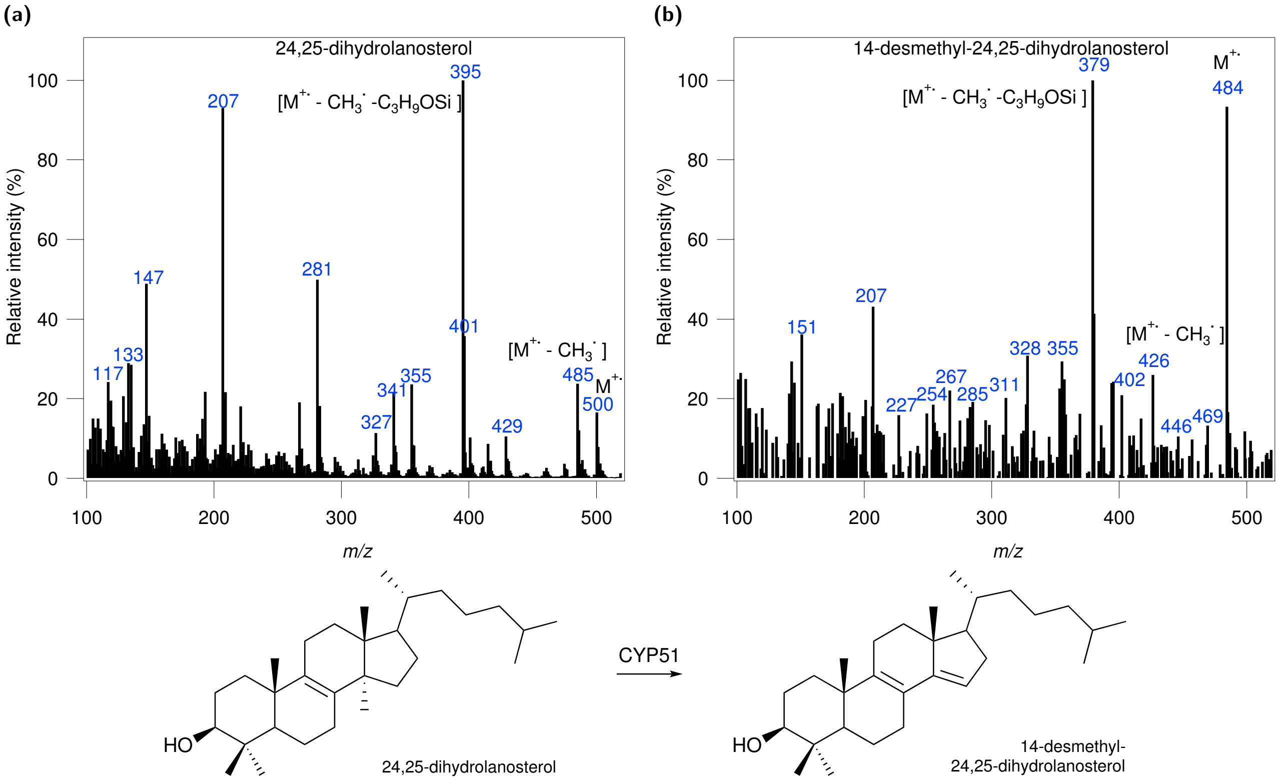

Figure S9 MS of (a) 24,25-dihydrolanosterol and (b) the single, new product observed in the turnover of CYP51*Dt* with 24,25-dihydrolanosterol. Starting material and product were derivatised as the 3-TMS ether before analysis by GCMS.

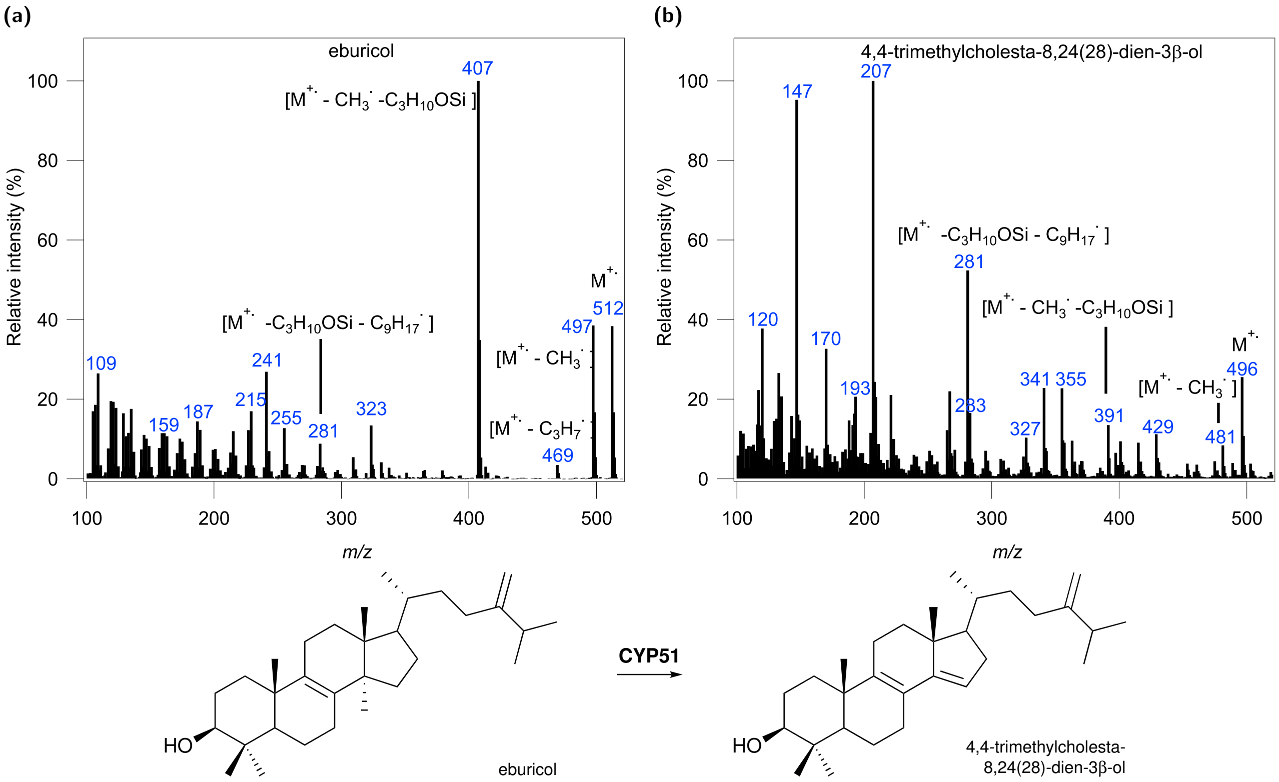

Figure S10 MS of (a) eburicol and (b) the single, new product observed in the turnover of CYP51*Dt* with eburicol. Starting material and product were derivatised as the 3-TMS ether before analysis by GCMS.

4.4 CYP51*Dt* substrate NMR

All NMR spectra were recorded at 500 MHz on a Bruker Avance 500MHz spectrometer. The $CDCl_3$ residual solvent peak was calibrated to δ_H 7.26 ppm.

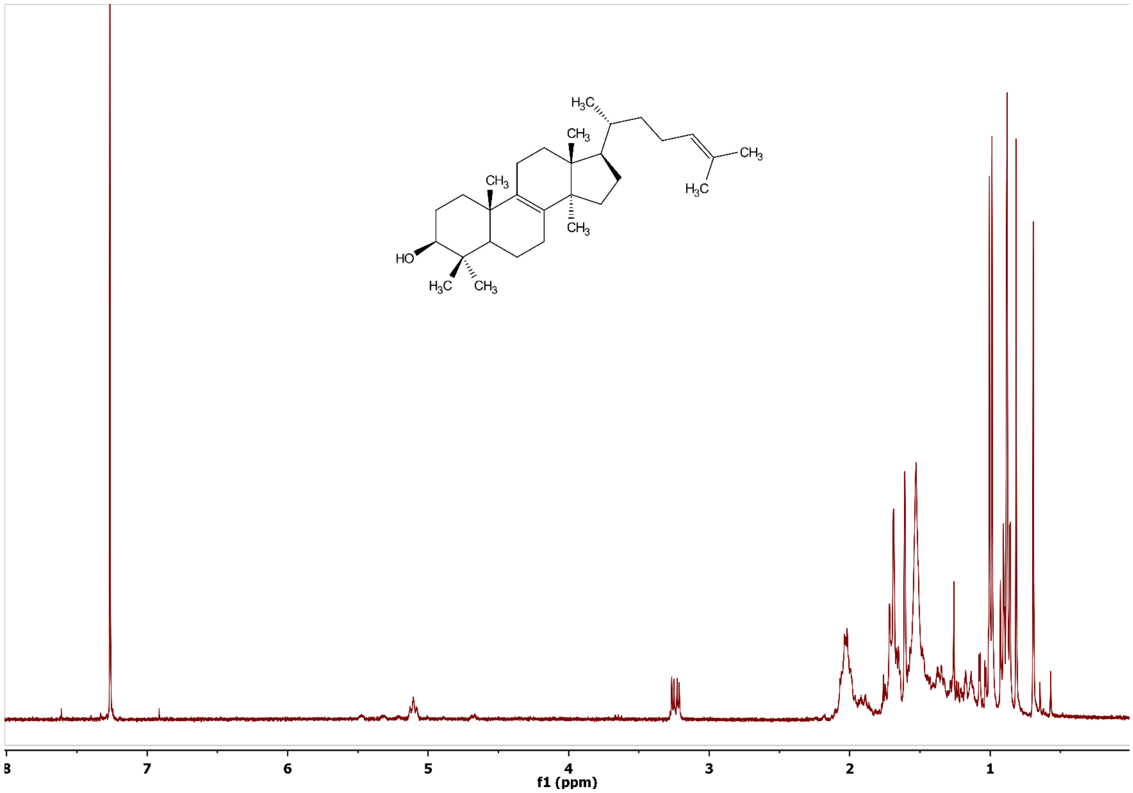

Figure S11 NMR spectrum of lanosterol (**6**).

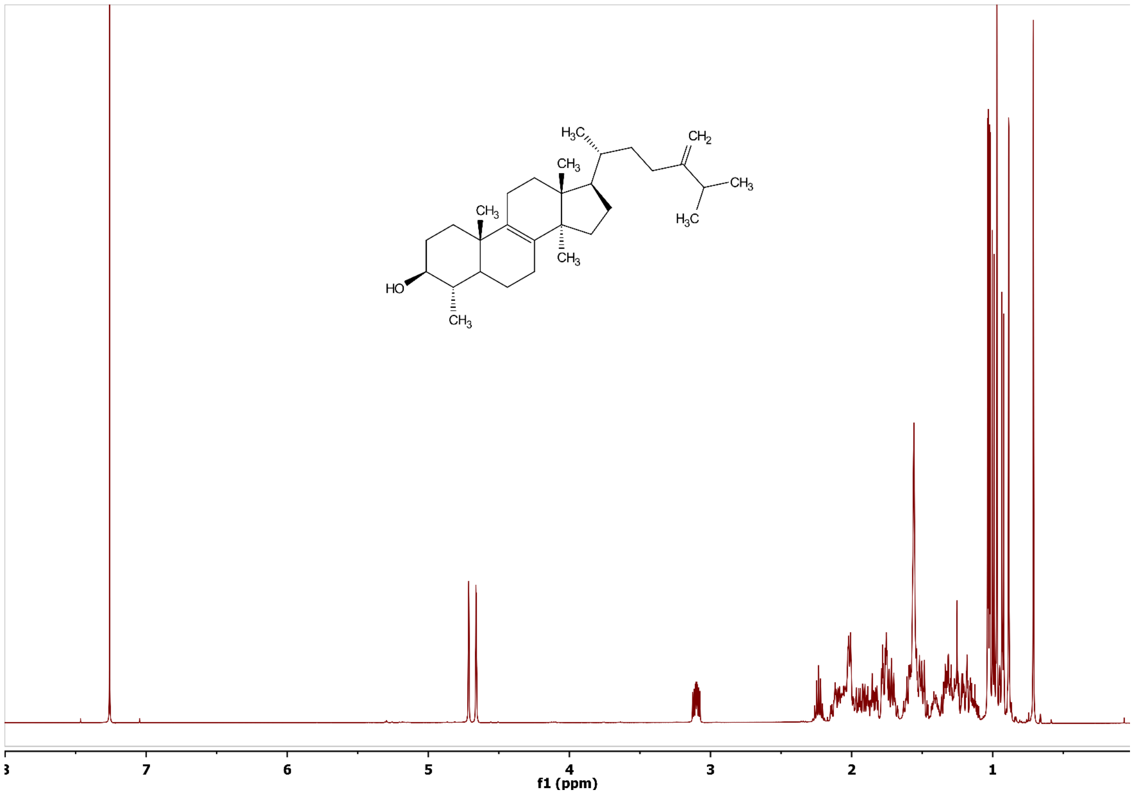

Figure S12 NMR spectrum of obtusifoliol (**7**).

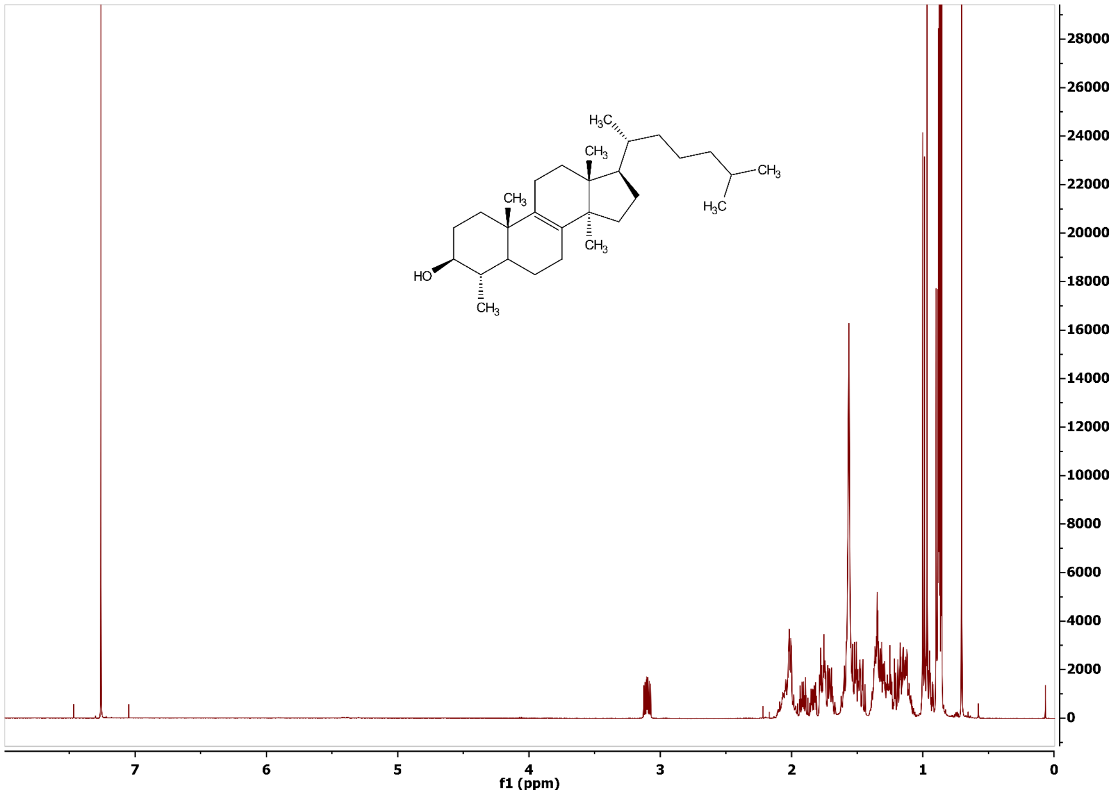

Figure S13 NMR spectrum of 4-desmethyl-24,25-dihydrolanosterol (**19**).

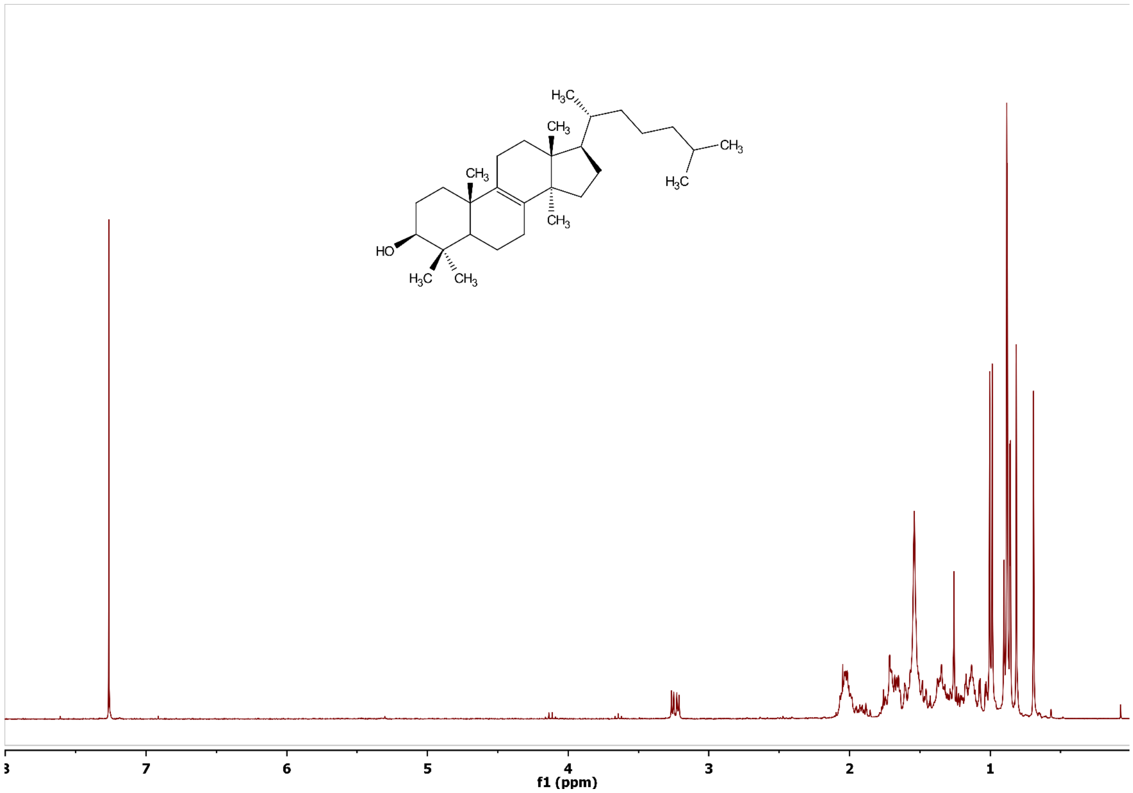

Figure S14 NMR spectrum of 24,25-dihydrolanosterol (**20**).

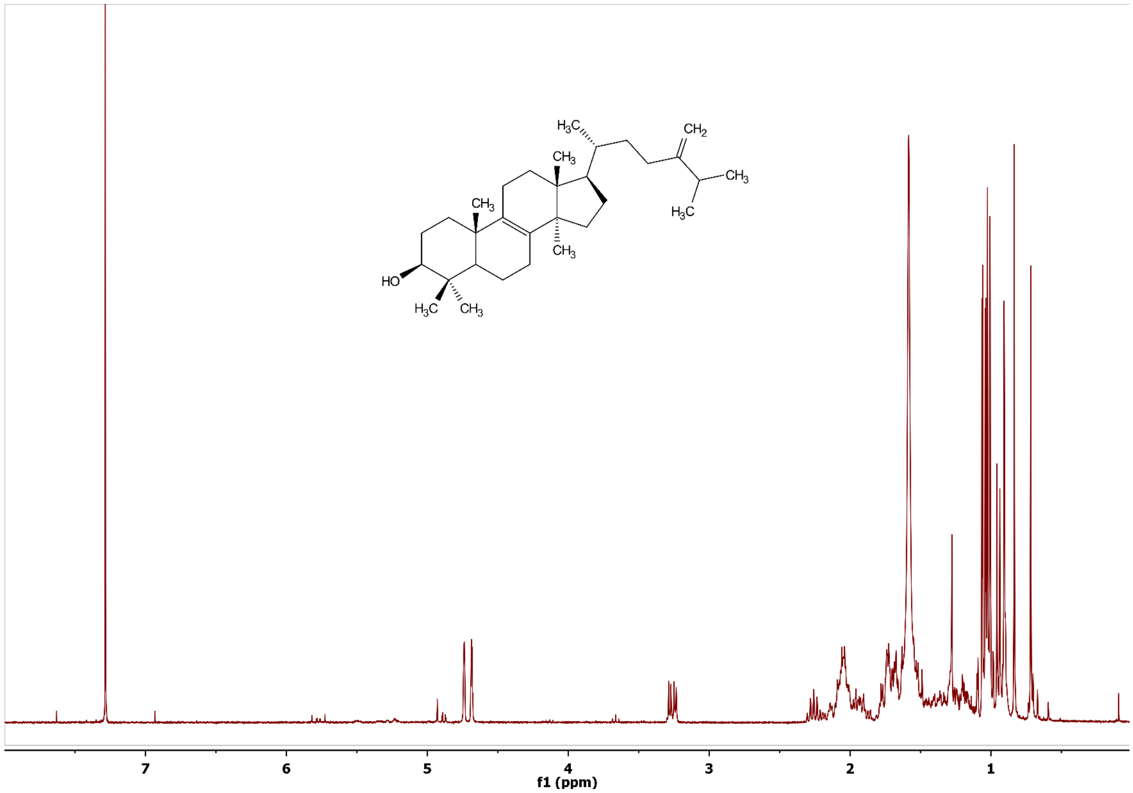

Figure S15 NMR spectrum of eburicol (**23**).

4.5 CYP51*Dt* SDS-PAGE gel

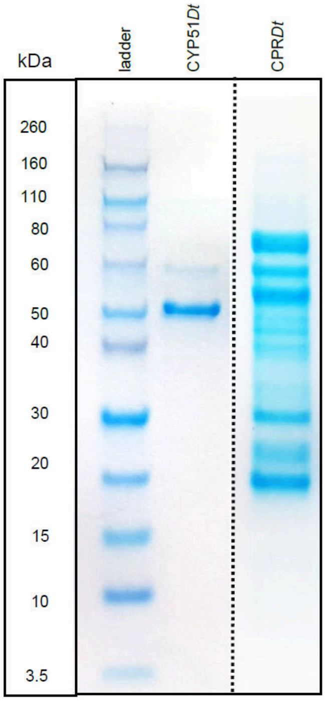

Figure S16 SDS-PAGE gel of purified CYP51*Dt* (calculated apoprotein 56.61 kDa, calculated to be >85% pure using densitometry) and CPR*Dt* (calculated apoprotein 77.34 kDa). Samples of CYP51*Dt* and CPR*Dt* were run on the same gel, intermediate lanes were deleted for clarity as indicated by the dashed black line.

Table S2 Matched peptides (shown in red) of fragment MS of CYP51*Dt*. Protein coverage 65%. Enzyme - Trypsin (cuts C-term side of KR unless next residue is P) Variable modifications - deamidated (NQ), oxidation (M).

1	MDLKENKFFS	IGLVFLATII	FIKLLAALFR	SGSKHRKPPI	VKSLPVIGGL
51	LRFMKGPILL	IRQEYQKLGS	VFTLNIVNRK	ITFFVGPEVS	SHFFKAPEVE
101	LSQQEVYQFN	VPTFGPGVVF	DVDYSVRQEQ	FRFFTESLRV	SKLKSYVDQM
151	VVEAEDYFSK	WGECGEVDLK	YELEHLIILT	ASRCLLGREV	RDKLFDDVSA
201	LFHDLDNGMI	PISVIFPYLP	IPAHRRRDRA	RARIAEIFST	IINSRKVSGK
251	SEDDMLQCFI	ESKYKDGRPT	TDGEITGLLI	AALFAGQHTS	SITSTWTGAY
301	LLRFKQYLAA	ALDEQKQLMK	KHGDKVNHDI	LAEMDVLYRC	IKEALRLHPP
351	LIMLLRQSHS	DFTVKTKEGI	EYDIPKGHIV	ATSPAFANRL	PYIYKDPDTY
401	DPDRFVPGRE	EDKVAGAFSY	ISFGGGRHGC	LGEPFAYLQI	KAIWTHLLRN
451	FEFELVSPFP	EIDWNAMVVG	VKGQVMVRYK	RRKLSVDSHH	HHHH

Table S3 Primers used for the isolation of genes from *D. transversa* cDNA for sequencing.

Primer	Sequence (5' { 3')	Primer	Sequence (5' { 3')
CPR*Dt*-AF	GACGGCCGGATCTATAGT	SSR*Dt*-R	TCTTTGCTAGTTGTGGAGATTGAGGATGTGCTTGA
CPR*Dt*-DR	CAATTTCGTTCCAGCG	51*Dt*-AF	TTCGATTTAAAAATCACACACA
SSR*Dt*-F	GCTTACCGACGGCGACGAGCTTCCA	51*Dt*-DR	TGCAGAATATATAAGGTAGATGG

Table S4 Primers employed to generate Δ*erg6 S. cerevisiae* and to confirm mutation.

Primer	Sequence
ERG6:H_F	GACATGGAGGCCCAGAATACCATAATTTAAAAAAACAAGAATAAAATAATAATATAGTAGGCAGCATAAG
ERG6:H_R	ATTCACTAGATCAATAAGATTCAAATAAAGCGCACGATATATACCTATTTGAATGCTGGTCGCTATACTG
ERG6:H_R	ATTCACTAGATCAATAAGATTCAAATAAAGCGCACGATATATACCTATTTGAATGCTGGTCGCTATACTG
SEQ_R	AGGGGAGCAGTCAATACGTT
SEQ_F1	AGGTTTTGAAACCGGGTGGT
SEQ_F2	CACTGGCTAAACATGCAGGC

Table S5 Primers employed to verify sequences.

Primer	Sequence (5' - 3')	Primer	Sequence (5' - 3')
CPR*Dt*-AF	GACGGCCGGATCTATAGT	51*Dt*-BR	GCTGAGATCTTTTCAACC
CPR*Dt*-AR	TTGTCCCTCAGTAAACCA	51*Dt*-CF	ATCCCTGCTCACCGC
CPR*Dt*-AR2	TGAAGCCAAACTCCC	51*Dt*-CR	CAAAGGGCCACATTGTT
CPR*Dt*-BF	CCGACTGATAATGCAGCTA	51*Dt*-DF	GACTTCACTGTGAAAACCA
CPR*Dt*-BR	AAAACACCAACATGATCTCC	51*Dt*-DR	CCATCTACCTTATATATTCTGCA
CPR*Dt*-CF	AATCTCTGGCACTGGAC	SSR*Dt*-AF	GACGAGCTTCCACTCCTT
CPR*Dt*-CR	GTCGCATTCTTCATCCA	SSR*Dt*-AR	CTTGTTAATATGGGCCAGAT
CPR*Dt*-CR2	TACAATCCTGATCTTCATCC	SSR*Dt*-BF	GATTACAAGCGTGCTAGACAT
CPR*Dt*-DF	TGGAAGGATTCACAAAGG	SSR*Dt*-BR	GCAATGATCTATACTCCGACT
CPR*Dt*-DR	CAATTTCGTTCCAGCG	SSR*Dt*-CF	GGTGAGGGGGAACTT
51*Dt*-AF	TTCGATTTAAAAATCACACACA	SSR*Dt*-CR	GGTGTATCCCATCTGGC
51*Dt*-AR	CAAGGCGCCTGAGGT	SSR*Dt*-DF	GTATCCGAAACTATTACCATGA
51*Dt*-BF	ATCACCTTTTTCGTTGG	SSR*Dt*-DR	GGTTGAAGTCTTCATCCTAGTT

Table S6 Primers employed to introduce restriction enzyme binding sites to *ssrDt*

Primer	Sequence
ssrDt_F1	ACCTGGATCCATGTCGGATCTTCGACTC
ssrDt_R1	CGATGTCGACCTAGTTATCCTCTGCATAGGCTTGTT

Table S7 Primers used to generate N- and C- terminal modifications for CPR*Dt* and CYP51*Dt*.

Primer	Sequence (5'-3')
CPR*Dt*-F1	TTATATCATATGAAACTCAGCGCGCTGGACTTA
CPR*Dt*-F2	TTATATCATATGGCGGTGGTGCTGCTGTTCTGGCGT
CPR*Dt*-R1	ATATAAAAGCTTGGTACCCTACCAAACATCACGCAA
CPR*Dt*H-R2	GAATTCAAGCTTTTAGTGATGGTGATGGTGATGCCAAACATCACGCAAATACCGGCCTT
51*Dt*-F1	TTATATCATATGGACTTAAAAGAGAACAAGTT
51*Dt*-F2	ATTCCATATGGCGAAAAAAAACCAGCAGCAAAGGCAAACTGCCACCTATCGTCAAATCACTTC
51*Dt*-R1	ATATAAAAGCTTGGTACCCTAACTATCAACACTC
51*Dt*H-R2	CTCGAATTCAAGCTTTTAGTGATGGTGATGGTGATGACTATCAACACTCAGCTTCCGAC
